# *Vitis labrusca* genome assembly reveals diversification between wild and cultivated grapevine genomes

**DOI:** 10.3389/fpls.2023.1234130

**Published:** 2023-08-31

**Authors:** Bo Li, Andrea R. Gschwend

**Affiliations:** Department of Horticulture and Crop Science, The Ohio State University, Columbus, OH, United States

**Keywords:** *Vitis labrusca*, *Vitis vinifera*, grapevine, comparative genomics, genetic variation, segmental duplication, zygosity, adaptive evolution

## Abstract

Wild grapevines are important genetic resources in breeding programs to confer adaptive fitness traits and unique fruit characteristics, but the genetics underlying these traits, and their evolutionary origins, are largely unknown. To determine the factors that contributed to grapevine genome diversification, we performed comprehensive intragenomic and intergenomic analyses with three cultivated European (including the PN40024 reference genome) and two wild North American grapevine genomes, including our newly released *Vitis labrusca* genome. We found the heterozygosity of the cultivated grapevine genomes was twice as high as the wild grapevine genomes studied. Approximately 30% of *V. labrusca* and 48% of *V. vinifera* Chardonnay genes were heterozygous or hemizygous and a considerable number of collinear genes between Chardonnay and *V. labrusca* had different gene zygosity. Our study revealed evidence that supports gene gain-loss events in parental genomes resulted in the inheritance of hemizygous genes in the Chardonnay genome. Thousands of segmental duplications supplied source material for genome-specific genes, further driving diversification of the genomes studied. We found an enrichment of recently duplicated, adaptive genes in similar functional pathways, but differential retention of environment-specific adaptive genes within each genome. For example, large expansions of NLR genes were discovered in the two wild grapevine genomes studied. Our findings support variation in transposable elements contributed to unique traits in grapevines. Our work revealed gene zygosity, segmental duplications, gene gain-and-loss variations, and transposable element polymorphisms can be key driving forces for grapevine genome diversification.

## 1 Introduction

Cultivated grapevine, *Vitis vinifera* subsp. *vinifera*, is grown in temperate climates and is economically significant worldwide. It has been domesticated from its wild Eurasian progenitor for the past 11,000 years to produce quality wine and table grapes (Zhou et al., 2017; Dong et al., 2023; Xiao et al., 2023). Due to the high heterozygosity of *V. vinifera* genomes, cultivated grapevines are commonly clonally propagated to maintain the genetics underlying desirable fruit characteristics (Franks et al., 2002; Riaz et al., 2002; Vondras et al., 2019). There are approximately 70 *Vitis* species that grow worldwide, and about 30 are native to North America and are adapted to diverse environmental conditions (Zecca et al., 2012; Liu et al., 2016; Ma et al., 2018; Walker et al., 2019). Wild grapevines have many desirable characteristics related to fruit quality and abiotic and biotic stress tolerance, making them important genetic resources for grapevine breeding and cultivar improvement (Staudt and Kassemeyer, 1995; Reynolds and Reisch, 2015). The historical use of wild *Vitis* species as rootstock to mitigate the devastation of European vineyards from grapevine pest, phylloxera, demonstrates the potential and importance of utilizing wild grapevine resources for sustainable viticulture (This et al., 2006; Myles et al., 2011). *Vitis labrusca*, which grows natively in the Eastern United States, is well known for its large berries, superior disease resistance, and cold tolerance (Fennell, 2004; Wang and De Luca, 2005; Cadle-Davidson, 2008; Cadle-Davidson et al., 2011; Yang et al., 2020). *V. labrusca*, and other wild grapevines, have been utilized in grapevine breeding programs to combine their adaptive traits with the fruit quality traits of *V. vinifera*, resulting in many successful interspecific hybrid cultivars, such as Concord (*V. labrusca* x *V. vinifera*) (Reynolds and Reisch, 2015; Wen et al., 2020). Although grapevine breeding programs have tapped into wild grapevine genetic diversity to produce resilient hybrid grapevines suitable for propagation in diverse environments, the genetics that underlie adaptive traits in wild grapevine, and how they evolved across *Vitis* genomes, are still largely unknown. The lack of a *V. labrusca* reference genome has slowed progress pinpointing the underlying genetics of these valuable traits.

The first fruit crop reference genome, published in 2007, was the PN40024 grapevine genome, a near homozygous genotype derived through self-crossing of *V. vinifera* cv. Helfensteiner, (a cultivar that originated from crossing cv. Pinot Noir and cv. Schiava grossa), which was generated using a whole-genome shotgun sequencing strategy (Jaillon et al., 2007; Velt et al., 2023). Since then, there have been significant advancements in long-read sequencing technology and genome assembly strategies, which have overcome the past challenges of assembling highly heterozygous genomes, improving the continuity of genomes and establishing two haplotype genomes, providing the opportunity to investigate intragenomic variation in diploid genomes (Minio et al., 2017). There are now reference-quality, haploid-resolved genome sequences available for *V. vinifera* cultivars and wild grapevine species (Chin et al., 2016; Roach et al., 2018a; Girollet et al., 2019; Minio et al., 2019a; Zhou et al., 2019; Patel et al., 2020; Wang et al., 2021; Zou et al., 2021). These data, along with the *Vitis labrusca* reference genome sequence we report here, provide the opportunity to uncover the intra- and intergenomic variations among Eurasian cultivated and wild North American grapevine species that contributed to the independent evolutionary trajectories of the grapevine genomes after divergence from a common ancestor ~47 million years ago (Mya) (Ma et al., 2018), and identify the genetic variation that facilitated their differential adaptation.

Segmental duplications (SD) can be a driving force for genome diversification and adaptation across species (Bailey et al., 2002; Marques-Bonet et al., 2009; Dennis and Eichler, 2016; Mérot et al., 2020). SDs create new genetic material, allowing duplicated genes to escape the selective constraints of their progenitor genes, which can lead to genetic novelty (Ohno, 1970; Matsuno et al., 2009). Preliminary studies of one hundred *V. vinifera* PN40024 BACs demonstrated SDs contribute significantly (~17.5%) to the grapevine genome (Giannuzzi et al., 2011). Indels and transposable elements (TEs) also contribute to genetic diversity, affecting zygosity and gene expression, which can contribute to new crop phenotypes and adaptation to local environments (Yan et al., 2006; Liu et al., 2008; Kanazawa et al., 2009; Chu et al., 2011; Schrader and Schmitz, 2019). Multiple traits of interest in grapevine are affected by indels in gene regulatory regions; for example, indels in the promoter region of the AMAT gene in *V. vinifera* varieties affected gene expression, resulting in reduced methyl anthranilate accumulation in the berries and loss of the “foxy” flavor that is characteristic of *V. labrusca* and its hybrid cultivar, Concord (Yang et al., 2020). Both insertions and deletions affecting the VvMybA1 genes influence grape berry skin color. For example, retrotransposon insertions in the VvmybA1 promoter region were found to contribute to the white-skinned berry phenotype in some grapevine cultivars and even a short insertion in the intron of the MybA1 gene was linked to reduced anthocyanin production, resulting in pink-skinned berries (Kobayashi et al., 2004; Shimazaki et al., 2011). Large structural rearrangements have also been found to affect berry color in grape, leading to hemizygosity of MybA genes in *V. vinifera* cultivars such as Chardonnay and Tempranillo (Zhou et al., 2019; Carbonell-Bejerano et al., 2017). In fact, chromosomal rearrangements have played a significant role in shaping the Chardonnay genome, leading to hemizygosity of ~15% of the genes (Zhou et al., 2019). By systematically annotating the structural variation within and across wild and cultivated grapevine genomes, we can discover the underlying genetics that contribute to the uniqueness of each grapevine species.

In this study, we generated a high-quality reference genome for *V. labrusca* and conducted comprehensive intragenomic and intergenomic analyses with three cultivated and one additional wild grapevine genome. We discovered that variation in SDs, gene zygosity due to indels, and TE insertion polymorphism in regulatory regions contribute to grapevine genome diversification and identified gene family expansions unique to the wild and cultivated grapevine genomes studied, which may contribute to unique traits.

## 2 Materials and methods

### 2.1 Plant materials

Cuttings of *Vitis labrusca*, Grem-4 (588583), a female plant, were requested from USDA-ARS. Grem-4 was selected based on its genetic classification as *V. labrusca* (Klein et al., 2018), its high resistance to pathogens, such as downy mildew (Cadle-Davidson, 2008), and its availability at the time of sequencing.

### 2.2 DNA extraction and PacBio sequencing

DNA was extracted from young leaves using a modified CTAB method. Briefly, 0.5g of *Vitis labrusca*, Grem-4 leaf tissue was grounded into fine powder with liquid nitrogen, 5 ml of pre-heated (65°C) CTAB extraction buffer was added, and the tube was incubated at 65°C for 30 mins. Two rounds of chloroform extractions were conducted to isolated DNA. 10 ul of RNase A was added and the tube was incubated at 37°C for 30 mins to completely remove RNA contamination. Finally, 95% ethanol was added to precipitate the genomic DNA. The quality and quantity were immediately checked with a 1.2% agarose gel, Nanodrop One (Thermo Fisher Scientific, US), and Qubit 4 Fluorometer (Thermo Fisher Scientific, US). The high-quality genomic DNA of *V. labrusca* was shipped to the University of Delaware DNA Sequencing and Genotyping Center for PacBio sequencing. Six PacBio SMRTbell libraries were prepared and sequenced with PacBio Sequel platform. A total of 58.46 GB of raw reads were generated with the subread N50 larger than 20.2 kb, totaling approximately 115x coverage of the *V. labrusca* genome (assuming an estimated genome size of 500 Mb). SequelQC was used to summarize and evaluate the sequencing results (https://github.com/ISUgenomics/SequelQC) (**Supplemental Table S1**).

### 2.3 Genome assembly and scaffolding

The overall genome assembly strategy can be viewed in **Supplemental Figure S1**. PacBio subreads longer than 10 kb were used for *de novo* assembly using Falcon_unzip (v1.2.0) with default parameters (Chin et al., 2016). The PacBio raw reads first were processed by self-correction and the resulting consensus reads were used to find overlap and then assemble into contigs. Because FALCON is a diploid-aware assembler, it produced a set of primary contigs (p-contigs) and a set of associate contigs (a-contigs) which represent divergent allelic variants. To improve the accuracy of contig assignment, we employed Purge Haplotigs (v1.1.1) (Roach et al., 2018b) to correct the contigs placed into wrong groups, which resulted in 376 p-contigs moved to the a-contig group based on alignment and coverage results. First, we ran the ‘hist’ function in Purge Haplotigs to determine the coverage and manually selected 15, 95, and 170 as cutoffs for the low, middle and high read depth. Second, we ran the ‘purge’ function to direct the contig assignment between the two haplotypes. We also developed a reference-based correction method to validate the results from Purge Haplotigs, illustrated in **Supplemental Figure S2**. We aligned all contigs onto the *V. vinifera* PN40024 reference genome sequence (12X.v2) using MUMmer4 (v.4.0) with “-maxmatch -l 100 -c 500” (Marçais et al., 2018) and grouped, ordered, and assigned all contigs into groups based on the PN40024 reference. For each contig from the p-contig group which was incorrectly assigned by Purge Haplotigs (i.e., should be placed into a-contig group), we generated a dot plot for manual inspection. Overlap between a single contig and the alignment of other ordered contigs (which represents the corresponding chromosomes in *V. labrusca*) further validated the contig was a genomic segment from that homologous chromosome. No overlap with ordered contigs resulted in the maintenance of the contig in the p-contig group. After this correction, we recalled 143 contigs from the previous Purge Haplotigs results, which eventually produced a primary assembly (p-contig) consisting of 438 contigs with a total genome size of ~502 Mb (**Supplemental Table S2 and S3**).

To scaffold contigs into pseudomolecules, we adopted a reference-based scaffolding method provided by RaGOO (v1.1) with “-t 10 -s -C” (Alonge et al., 2019). We aligned contigs onto the PN40024 reference to separate all contigs into groups corresponding to the 19 V*. labrusca* chromosomes. The contigs that aligned to the same PN40024 chromosome were ordered and joined to form continuous pseudomolecules with 100 “N's” representing gaps between two neighboring contigs. Finally, this resulted in 19 V*. labrusca* pseudomolecules and two other contig sets (one corresponded to PN40024 unassembled contigs and the other consisted of *V. labrusca* contigs which had no homologous sequences in the PN40024 reference). Dot-plots between the *V. labrusca* genome and PN40024 were generated by MUMmer4. Manual inspection was used to resolve complex regions of the assembly for sequence continuity and accuracy, e.g., contig order and redundant overlapping regions between two neighbor contigs. For downstream analyses, if not specifically mentioned, the primary assembly is used as the *V. labrusca* reference genome.

### 2.4 Centromeric repeat identification

*V. vinifera* centromeric repeat sequences were retrieved from a previous publication (Di Gaspero and Foria, 2015). A 107-nucleotide monomer (AGTACCGAAAAAGGGTCGAATCAGTGTGAGTACCGAAAAATGGTAGAATCCGGGCGAGTACCGGGAAAAGGTAGAATCCGTGCGAGTATCGAAAAACTGTCCGGGCG) was used to search the entire *V. labrusca* genome using BLASTN (Altschul et al., 1997). BLAST hits (>90% identity and >95% coverage) were used to define the centromere/pericentromeric regions of the *V. labrusca* genome.

### 2.5 Genome annotation

To identify transposable elements (TEs), several *de novo* TE discovery tools were used to find intact TEs based on their structure signatures. We adopted LTR_retriever (v2.8) (Ou and Jiang, 2018) to identify Long Terminal Repeat retrotransposons (LTR-RTs) in the *V. labrusca* genome and MITE-Hunter (Han and Wessler, 2010) for Miniature inverted-repeat transposable element (MITE) discovery. Helitron transposons were screened by running HelitronScanner (Xiong et al., 2014). Furthermore, a genome-specific TE repeat database was built by running RepeatModeler (v2.0) (http://www.repeatmasker.org/RepeatModeler) with *de novo* repeat finding programs: RECON (v1.08) and RepeatScout (v1.0.6) with default parameters. To generate the final repeat databases for *V. labrusca*, we combined the results above after removing the overlapped exemplars. This repeat database was then used to mask the entire genome of *V. labrusca* by running RepeatMasker (v4.1.0) (http://www.repeatmasker.org). For the comparative analysis, we also applied the same strategy to identify TEs within the other grapevine genomes studied.

The RepeatMasked genome was used to annotate protein coding genes using MAKER (v2.31.10) (Campbell et al., 2014). Both Augustus (v3.3.2) (Stanke et al., 2008) and SNAP (Version 2006-07-28) (Korf, 2004) were used as *de novo* gene predictors to run MAKER. We used Arabidopsis-trained parameters to predict genes in the *V. labrusca* genome to get a first set of parameters. Then, we ran MAKER with these parameters along with supporting evidence from RNA-seq (PRJNA389437), Iso-seq (PRJNA433195), and PN40024 reference protein sequences (downloaded from Ensembl v43). Only gene models with AED=0 (gene model had 100% support from external evidence) were maintained. 573 gene models with multiple exons, were used to train Augustus again and MAKER was run again with the same expression and protein evidence. The protein sequences were aligned to the protein databases using InterProScan (Jones et al., 2014). The resulting gene annotations were filtered based on either having an InterProScan hit or MAKER AED>0.5, which generated the final version of the *V. labrusca* gene annotation. The final gene dataset was assessed by running BUSCO (v3.0.1) (Seppey et al., 2019) and BLASTP search (1e-5) with PN40024 protein sequences.

Non-coding RNA (ncRNA) were searched genome-wide using Infernal (Nawrocki and Eddy, 2013). For ribosomal RNA (rRNA), BLAST was conducted with Arabidopsis rRNA sequences as the query to search against the *V. labrusca* genome and other available grapevine genomes, including *V. vinifera* Cabernet Sauvignon (Chin et al., 2016), *V. vinifera* Chardonnay (Roach et al., 2018a), PN40024 (Canaguier et al., 2017), and *V. riparia* (Girollet et al., 2019).

### 2.6 Large scale structural variations between *V. vinifera* and *V. labrusca*

Large scale SVs were observed between PN40024 and *V. labrusca* based on a dot plot generated by MUMmer4. For the alignments, we used contig sequences from *V. labrusca* as a query to detect the SVs within contigs to avoid the biases of the reference-based scaffolding strategy. Besides SV calling from the alignment approach, we also carried out an alignment-free approach by using smash++ (v20.04) with “-l 0 -m 1000” (Hosseini et al., 2020). To further validate these large SVs, we conducted SV analyses between *V. labrusca* and the Cabernet Sauvignon, Chardonnay, and *V. riparia* genomes following a similar approach as we did for PN40024 and *V. labrusca*. For each SV we identified between *V. labrusca* and the PN40024 reference, we checked whether it was validated with other genomes. Only the SVs that were supported by multiple genomes were included here as potential SVs. In addition, read alignments were also applied to validate these SVs. Genome ideogram figures were drawn with RIdeogram (Hao et al., 2020). For these and the other comparative genomic analysis detailed below, we accessed the genome data for the four other grapevines here: PN40024 (http://plants.ensembl.org/index.html), *V. vinifera* Cabernet Sauvignon(https://cantulab.github.io/data.html), *V. vinifera* Chardonnay (https://zenodo.org/record/1480037#.X-OhIelKhRE), and *V. riparia* (Vrip) (https://www.nature.com/articles/s41597-019-0133-3#Sec9).

### 2.7 Intra-genomic variations analysis between homologous chromosomes

To estimate the overall heterozygosity in the *V. labrusca* genome, we aligned associate genome sequences onto primary sequences on one chromosome-to-chromosome mode by using *dnadiff* script wrapped within MUMmer4. For each chromosome pair alignment, we collected two sources of sequence differences from the alignment results: sequences without any alignments (representing unique sequences in the associate genome) and dissimilar bases within alignments (divergent genomic regions between two homologous chromosomes). The heterozygosity was calculated using both the unaligned bases and the dissimilar bases between aligned sequences for each chromosome and the entire genome.

We defined three kinds of genetic variations: Structural Variations (SVs) (>30bp), Small Indels (2-30 bp), and Single Nucleotide Polymorphisms (SNPs) between the primary and associate assemblies in *V. labrusca* (30bps was chosen as the threshold separating SVs and Small Indels based on the NGMLR software default parameters). To characterize SVs and small Indels between the two sub-genomes (primary and associate assemblies), the 40x self-corrected PacBio long reads generated with Canu (Koren et al., 2017) were aligned on the primary assembly of the *V. labrusca* genome with NGMLR (v0.2.7; minimum SV length of 2 and default parameters) (Sedlazeck et al., 2018), and SVs were called by Sniffle (v1.0.11) (Sedlazeck et al., 2018). We ran LongShot (v0.4.1) with default parameters (Edge and Bansal, 2019) to call SNPs between homologous chromosomes. After filtering low quality SNPs, we kept heterozygous SNPs for downstream analysis. We applied SnpEff (v4.3t) (Cingolani et al., 2012) to functionally annotate genes potentially impacted by the SNPs.

### 2.8 Classification of hemizygous, heterozygous and homozygous genes

We classified each gene in the *V. labrusca* genome as either hemizygous, heterozygous, or homozygous based on SV results. Hemizygous genes were completely missing from one of the haplotypes due to deletions called by NGMLR+Sniffle pipeline. First, self-corrected PacBio long reads (comprising both the pseudomolecules and the unplaced contigs) were aligned on the primary genome to call deletions. Then we detected if a gene was situated within the deletion. The read alignments in genomic regions over a hemizygous locus showed two types of alignments. The first was reads that spanned the gene locus, meaning these reads were from the subgenome having the allele (the gene was present in the reads). The second was split reads mapping onto the boundary of the locus, but not spanning the hemizygous locus, which represents the reads from the other subgenome that lost this allele. The 2,048 gene sequences located in unplaced contigs were extracted and aligned to all hemizygous genes to verify no homologs to the hemizygous genes were present in unaligned contigs, and none of these genes showed high homology with the hemizygous genes. Hemizygous genes were also randomly selected for Integrative Genomics Viewer (IGV) inspection (Robinson et al., 2011). Both the reads covering this gene locus (one allele present) and split reads in this locus (one allele missing) can be observed. Heterozygous genes were determined at the gene-level and protein-level. Generally, if SV, small indels, or SNPs, caused deletions, insertions, or nucleotide changes within the gene body (exons and introns) between the two alleles, it was a gene-level heterozygous gene. If SV, small indels, or SNPs in exons caused a change in the amino acid sequence between two alleles, the gene was defined as a protein-level heterozygous gene.

### 2.9 Collinearity analysis among *V. vinifera*, *V. riparia* and *V. labrusca*

Gene collinearity analysis was conducted with MCScanX (Wang et al., 2012) across the five grapevine genomes (primary assemblies if the genomes were phased), including three cultivated grapevine varieties, grapevine reference genome PN40024, *V. vinifera* Cabernet Sauvignon (Cab), and *V. vinifera* Chardonnay (Char), and two wild North American grapevine species, *V. labrusca* (Vlab) and *V. riparia* (Vrip). First, we performed all-against-all alignments with all protein sequences from the above genomes with DIAMOND (v0.9.29.130) with the parameters “-e 1e-4 –max-target-seqs 1 –unal 1 -f 6” (Buchfink et al., 2015). Second, the bed files recording the genomic positions for all genes were extracted from corresponding GFF3 files. Third, we fed the alignment output files and bed files to MCScanX, using default parameters, to establish gene collinearity. Finally, we filtered out the redundant collinear blocks by E-values (lower E-value was maintained). For collinearity analyses, we either used PN40024 or *V. labrusca* as the reference genome and classified the gene models into different groups: All Shared, Wild Shared, Cultivated Shared, and Genome specific.

### 2.10 The origin of hemizygous genes in Chardonnay

Chardonnay was proposed to be a cross between two very different *V. vinifera* cultivars: Pinot Noir and Gouais blanc. Non-collinear genes in parental plants should lead to the inheritance of hemizygous genes in hybrids. We used the reference genomes from Chardonnay and PN40024 (representing Pinot Noir), to determine if there were collinear genes in the PN40024 genome for each of the hemizygous genes in the Chardonnay genome, based on established gene collinearity (Materials and Methods 2.9). PN40024 is not a direct parent of Chardonnay, but it shares some genetic background with Pinot Noir and was available at the time of the analysis. Next, we investigated the presence and absence of these Chardonnay hemizygous genes in Gouais blanc WGS data (60x Illumina reads) (NCBI, SRA : SRR7188231) (Roach et al., 2018a). Because Gouais blanc lacked a reference genome, we applied a mapping strategy to roughly estimate the PAV of hemizygous genes in Gouais blanc (but this approach does not provide collinearity information). This approach may overestimate the discovery rate of collinear genes between Chardonnay and Gouais blanc because of the existence of non-collinear duplicates. To lower the false positive rate, we mapped the Gouais blanc Illumina paired-end reads onto the Chardonnay genome and defined the genes as present, only if the gene was fully covered by the Gouais blanc reads.

### 2.11 Functional annotation of protein coding gene dataset

We aligned the protein sequences for all of our *V. labrusca* annotated genes to the UniprotKB database (https://www.uniprot.org/) using DIAMOND with default parameters, establishing homology to Uniprot proteins, and assigned GO terms to each gene in the *V. labrusca* genome. The Gene Core ontology file was download from (http://purl.obolibrary.org/obo/go.obo). PN40024 protein sequences were downloaded from Ensembl. In addition, we generated protein sequence data for Cabernet Sauvignon, Chardonnay, and *V. riparia* based on both genome sequences and gene annotation. CDS were first extracted from the genomes according to GFF3 files, and then were translated into protein sequences. Raw protein sequences were subsequently filtered by removing protein sequences that did not start with ATG (start codon). Using the same approach as we did with the *V. labrusca* protein sequences, we conducted GO annotation for the other four grapevines. GO enrichment analysis was conducted with R package, ClusterProfiler (v3.10.1) (Yu et al., 2012). We constructed GO datasets for each grapevine genome, p-adjusted values were calculated by Benjamin-Hochberg correction method, and 0.05 was selected as the cutoff for enrichment analysis.

### 2.12 Segmental duplication analysis

We conducted a Whole Genome Assembly Comparison (WGAC) to identify segmental duplications (SDs) in the five grapevine genomes. Genomic sequences from the 19 chromosomes for each of the five grapevine genomes (primary assemblies) were prepared for pairwise MUMmer alignment with “–maxmatch and –nosimplify” parameters. For each genome, the resulting 342 inter-chromosome MUMmer alignment output files were used to extract potential SDs by setting > 1 kb and > 90% as cutoff values based on the definition of SDs (Bailey et al., 2002). We collected all SDs with full gene coverage to study gene evolution via SD. Based on the collinearity among the five grapevine genomes, we identified genome-specific SD genes. Both CDS and protein sequences for the SD genes were obtained and aligned with Muscle (v3.8.1) with the “-clw” parameter and was converted into phylip format by a custom Perl script. The script “pal2nal.pl” was then run with parameters “-nogap” to generate input files for Ka/Ks calculation. The Ka/Ks ratios between SD genes were calculated by using codeml in PAML (Yang, 2007). The codeml parameters were: runmode= -1, seqtype=1, CodonFreq=0, kappa=1 and omega=0.5. Genome-specific duplicated genes were annotated with KEGG (Kanehisa et al., 2016) and GO for functional enrichment analysis. KEGG analysis was performed using KOBAS 3.0 (http://kobas.cbi.pku.edu.cn).

### 2.13 Gene family evolution

Orthologous groups were identified using the protein sequences (from the primary assemblies) of all five genomes by running OrthoFinder (v2.3.10) with default parameters (Emms and Kelly, 2019). The OrthoFinder results were processed and ClusterVenn (within OthoVenn2 online version) was applied to produce the Venn diagram figure (Xu et al., 2019). A set of 665 single copy orthologous genes across the five grapevine genomes and two outgroups, Arabidopsis and Poplar, were used to produce a maximum likelihood (ML) species tree using FastTree (v2.1.10) (Price et al., 2010). Gene family contraction and expansion patterns were analyzed with CAFE5 (v1.0) (De Bie et al., 2006). The ultrametric, rooted tree in Newick format was generated using r8s (v1.81) based on the ML tree (Sanderson, 2003). Significant rapidly evolved gene families in each species, and between cultivated and wild species, were further annotated with gene ontology (GO) terms.

### 2.14 NLR gene family evolution

Protein sequences from the five grapevine genomes were used to identify conserved NLR domains NB-ARC, canonical N terminal domains TIR (Toll/interleukin-1 receptor), CC (coiled-coil), CC_R_ (RPW8-like coiled-coil), and C terminal domain LRR (leucine-rich repeat) by using InterProScan with the pfam and coil databases (Jones et al., 2014). We defined NLR genes based on methods previously used in Arabidopsis (Van de Weyer et al., 2019). Identified NLR genes were classified into 4 subgroups based on the signature domains: TNLs (TIR domain), CNLs (CC and NB-ARC domains), RNLs (CC_R_ domain), and NLs (NB-ARC only or with LRR domains). The integrated domains (IDs) within each NLR were also annotated based on InterProScan results and compared between grapevine species, especially between cultivated and wild grapevines. NLR gene clusters were determined by adopting the definition that clustered genes were located within 200 kb genomic regions (Van de Weyer et al., 2019) and were plotted across 19 chromosomes. Previously published *P. viticola* resistance QTLs (RPV loci) were collected from (https://www.vivc.de) and assigned to the *V. labrusca* reference genome based on collinearity with PN40024 gene regions. NLR protein sequences were first aligned using MAFFT (v7.455) (Katoh et al., 2002), the poor-quality alignments were then trimmed by TrimAl (v1.2.59) (Capella-Gutiérrez et al., 2009), and a phylogenetic tree was computed by FastTree (v2.1.11) (Letunic and Bork, 2019) with JTT+CAT model and displayed with iTOL.

### 2.15 RNA-sequencing and expression analysis

RNA-seq data from grape whole berry tissue (from pre-veraison to veraison) for different grapevine species/varieties were retrieved from NCBI, including Pinot Noir (PRJNA260535), Chardonnay (PRJNA260535), Cabernet Sauvignon (PRJNA433195), and *V. labrusca* (PRJNA606742). Iso-seq data from berries of Cabernet Sauvignon was also downloaded from NCBI SRA (PRJNA433195) (Minio et al. 2019b). Four replicates for Cabernet Sauvignon and Chardonnay, and three replicates for Pinot Noir and *V. labrusca*, were used. Raw RNA-seq data was first checked for quality by FASTQC (v0.11.9) (Andrews, 2018). Adapter sequences and low-quality bases were trimmed using Trimmomatic (v0.35) (Bolger et al., 2014) with parameters:*SLIDINGWINDOW:4:5, LEADING:5, TRAILING:5, MINLEN:50*. The clean data were mapped onto reference genomes by HISAT2 (v2.1.0) (Kim et al., 2019) and gene expression was quantified by StringTie (v1.3.6) (Pertea et al., 2015). Also, raw counts were called by using HTseq (v0.11.1) (Anders et al., 2015). The raw counts from HTseq for the collinear genes across the grapevine genomes (Materials and Methods 2.9) were used by DESeq2 (v1.18.1) (Love et al., 2014) to determine the differentially expressed genes among different grapevine genomes.

### 2.16 TE insertion polymorphism and gene regulation

For LTR and MITE insertion polymorphism, we first compared the insertion polymorphism around collinear genes, within 1 kb upstream, gene body, and 1 kb downstream regions. We defined TE insertion polymorphisms as having no TE insertion in at least one genome (e.g., TE proportion in 1 kb upstream is 0), but in at least one genome, there is ≥200bp (20%) TE insertion in the 1 kb upstream. For detecting potential regulation of TEs on gene expression, we combined the LTR and MITE proportion first, and drew correlations between the total proportion of TE in the 1 kb upstream region and normalized gene expression values (log_2_(TPM+1)). Correlation coefficients lower than -0.9 were initially collected as potential genes whose expression may be regulated by TE polymorphism. We chose VvMybA1 and AMAT genes to demonstrate the connection between gene expression and TE insertion. Genotyping was performed on 1 kb upstream regions of VvMybA1 and AMAT genes in the Chardonnay, Cabernet Sauvignon, and *V. labrusca* genomes. First, the gene sequences and 1 kb upstream regions were extracted from both primary and associate assemblies. Then, multiple sequence alignments were conducted to compare the sequence similarity of 1 kb upstream regions between two alleles.

## 3 Results

### 3.1 *V. labrusca* genome assembly and annotation

A total of 58.46 Gb of PacBio reads (subread N50 = 20.3 kb) were generated and assembled into a reference genome for *V. labrusca* (**Supplementary Table S1**). The genome was phase assembled into primary and associate contigs, differentiating the two haplotypes making up the diploid *V. labrusca* genome, using Falcon-unzip (Chin et al., 2016) (**Supplementary Figure S1**). The primary contigs represent the near-complete genome, whereas the associate contigs represent the heterozygous portions of the genome, which vary in sequence from the primary assembled contigs. Next, contigs assigned to the primary and associate assemblies were assessed for accuracy using Purge Haplotigs, and PN40024-alignment validation was further utilized to identify 143 misplaced contigs, which were relocated to their appropriate assembly (Roach et al., 2018b; See Methods and **Supplementary Figure S2**). Subsequently, we generated a primary assembly (Vlab_PG) consisting of 438 contigs (Contig N50 is 2.5 Mb) with a total length of 502 Mb, close to the 507 Mb/C genome size detected by flow cytometry (Lodhi and Reisch, 1995), and an associate assembly (Vlab_AG) of 404 Mb with 2,077 contigs (**Supplementary Table S2**). Finally, we employed a reference-based scaffolding strategy, by aligning the *V. labrusca* primary assembly to the *V. vinifera* PN40024 grapevine reference genome (Jaillon et al., 2007; Canaguier et al., 2017), to build 19 pseudomolecules (456 Mb, 322 contigs) and 116 unassembled contigs (45.77 Mb), to establish a chromosome-level *V. labrusca* reference genome (**Supplementary Figure S1** and **Supplementary Table S3**).

**Figure 1:**
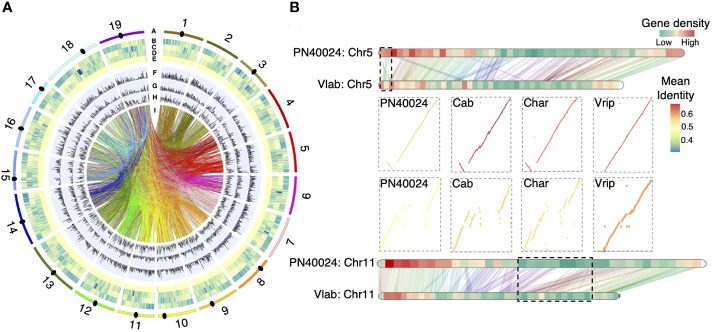
Grapevine genome annotation and large structural variations. **(A)** Circos plot displaying the genomic features of *V. labrusca*. From outside to inside, A) pseudomolecules (primary assembly), B) gene density, C) RNA-TE density, D) DNA-TE density, E) ncRNA density (100 kb window), F) intragenomic SNPs, G) intragenomic insertions, H) intragenomic deletion (counts per 100 kb window), and I) segmental duplication events (only SDs including genes were shown). The black ovals indicate the relative position of centromeres that could be identified in 14/19 pseudomolecules. **(B)** Validation of large structural variations between *V. labrusca* and *the PN40024 reference*. Large structural variations were first identified between *V. labrusca* and PN40024 and then validated by the other three genomes. One inversion on Chr5 and one indel on Chr11 (bordered by black dashed lines) were validated by multiple genome comparisons. (PN40024; Cab, *V. vinifera* Cabernet Sauvignon; Char, *V. vinifera* Chardonnay; Vlab, *V. labrusca*; Vrip, *V. riparia*).

We annotated 35,915 putative protein coding genes, which covered 97.1% of the BUSCO genes (**Supplementary Figure S3**). Gene density varies greatly across the chromosomes from telomere to centromere, e.g., the gene density of chromosome 5 varied from 40 to 91 genes/Mb (**Figure 1A**). Approximately 49.8% of the *V. labrusca* genome was comprised of repetitive sequences, consisting of 26.9% LTR RNA retroelements, with Gypsy LTRs being most abundant (12.3%), and 7.7% DNA transposons, with DTM (Mutator) (2.2%) and DTT (Tc1/Mariner) (3.1%) being most common (**Supplementary Table S4**). We investigated the non-coding RNA in the *V. labrusca* genome and predicted 314 miRNAs, 293 snoRNAs, 125 snRNAs, and 667 tRNAs (**Figure 1A**). We also located one 5S rRNA gene cluster (49 copies) on Chr17 and two 45S rRNA gene clusters on Chr15 and Chr19 (**Supplementary Table S5**). By using conserved centromere repeat sequences from *V. vinifera*, 14 of the 19 centromeric regions were also located (**Figure 1A**).

Overall, high collinearity was observed between the *V. labrusca* (Vlab) and the *V. vinifera* PN40024 genomes (**Supplementary Figure S4**), yet 22 large structural variations (SVs) (occurring within contigs) were detected, especially in gene-poor regions (**Figure 1B**; **Supplementary Figure S5**). To further validate these large SVs, we compared *V. labrusca* with two additional cultivated grapevine genomes, *V. vinifera* Cabernet Sauvignon (Cab) (Chin et al., 2016) and *V. vinifera* Chardonnay (Char) (Roach et al., 2018a), and one wild North American grapevine species genome, *V. riparia* (Vrip) (Girollet et al., 2019), which were selected based on the availability of reference-quality genomes at the start of this study. Three inversions (Chr5, Chr12 and Chr17) and one indel (Chr11) between cultivated and wild grapevines were validated by multiple genome comparisons (e.g., differences in indel presence/absence on Chr11 was consistently seen between *V. labrusca* and three cultivated grapevine genomes, but *V. labrusca* and *V. riparia* both shared this indel) (**Figure 1B** and **Supplementary Figure S5**). Several genome-specific SVs were also observed, but further support is needed to determine if they are true genome-specific SVs or if they were caused by assembly errors (**Supplementary Figure S5**).

### 3.2 Extensive intragenomic structural variations contribute to high haplotype variation in the *V. labrusca* genome

Cultivated grapevines have been proposed to be highly heterozygous, due to hybridization of diverse genotypes, subsequent clonal propagation, and the accumulation of somatic mutations (Velasco et al., 2007; Vondras et al., 2019). To investigate the extent of intragenomic sequence variation in cultivated and wild grapevine genomes, we conducted direct sequence comparisons between the primary and associate contig sequences of four grapevine reference genomes, wild *V. labrusca* and *V. vinifera* ssp. *sylvestris* (Massonnet et al., 2020) and cultivated Chardonnay and Cabernet Sauvignon, using the *dnadiff* function in MUMmer4 (Marçais et al., 2018). Alignments revealed the overall intragenomic variation of *V. labrusca* (including both unaligned bases and dissimilar bases between the two sub-genomes) was 4.99%, with great variation (2.19% to 8.78%) between individual chromosomes (**Supplementary Table S6**). The haplotype variation for *V. vinifera* ssp. *sylvestris*, a wild European grapevine, was 7.83%, whereas the haplotype variation for the two cultivated grapevine genomes assessed, Chardonnay and Cabernet Sauvignon, were 10.08% and 16.98%. Even though genome assembly quality could affect these results to some degree, we found the cultivated grapevine genomes studied here had higher overall intragenomic variation compared to the wild species (**Supplementary Table S7-S9**), likely reflecting the hybridization of more diverse parental genotypes and subsequent clonal propagation, to maintain such diversity (Bowers et al., 1999).

**Figure 2:**
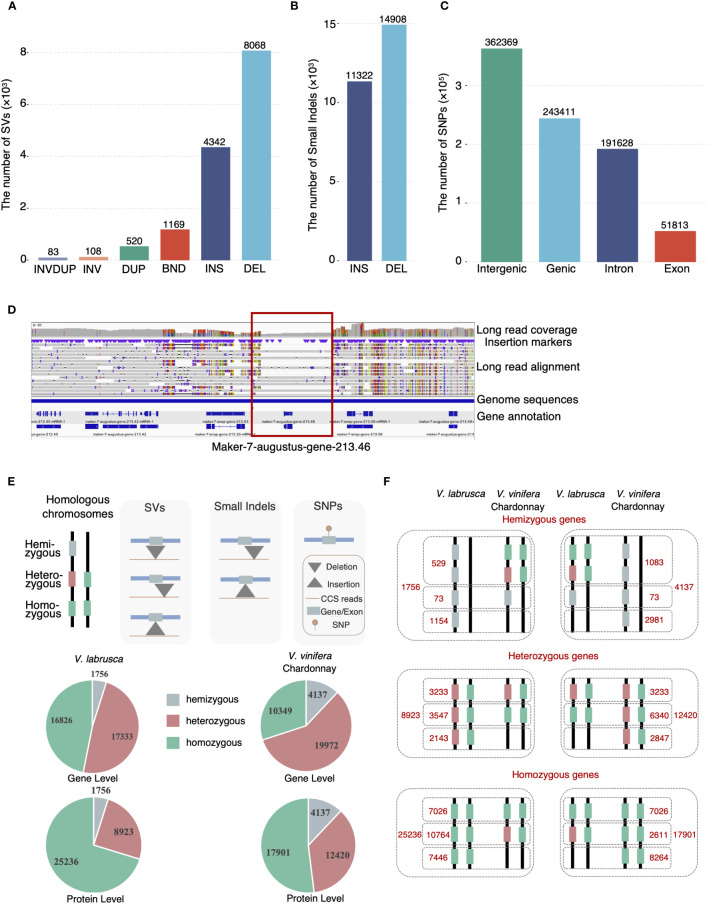
Intra-genomic variations of the *V. labrusca* and *V. vinifera* Chardonnay genomes. **(A)** The number of each type of intra-genomic structural variations identified in *V. labrusca*. DEL, deletion; INS, insertion; BND, Breakend; DUP, duplication; INV, inversion; INVDUP, inversed duplication. **(B)** The number of insertion (INS) and deletion (DEL) small indels between *V. labrusca* homologous chromosomes. **(C)** SNP distribution within different genomic regions in *V. labrusca*. **(D)** A snapshot of Integrative Genomics Viewer (IGV) depicting an example of a hemizygous gene (gene-213.46 within the red rectangle) identified via the mapping approach. Sequences for this gene are only present in half the reads aligned to this locus (shaded gray bars in the “Long read alignment” region). **(E)** Characterization of gene zygosity. Hemizygous and heterozygous genes can be identified by SVs, small indels, and SNPs. Variations occurring within coding regions that change the amino acid sequences are defined as “protein level” heterozygous genes and “gene-level” heterozygous genes have variations in the nucleotide sequence that may or may not affect the amino acid sequence. **(F)** Gene zygosity comparisons for *V. labrusca* (left panels) and *V. vinifera* Chardonnay (right panels). All genes in both genomes were classified as either hemizygous (top panel), heterozygous (middle panel), and homozygous (bottom panel). Collinear genes and non-collinear genes were determined between the *V. labrusca* and Chardonnay genomes and of the collinear genes, a comparison of gene zygosity was further determined for each genome, with the breakdown of each total listed. Hemizygous genes are denoted as gray squares, heterozygous as one green and one red square, and homozygous as two green squares.

To further investigate how different types of sequence variation contribute to the intragenomic differences observed between the *V. labrusca* haplotypes, we established a comprehensive pipeline, using the *V. labrusca* primary assembly as the reference, to identify SVs (>30bp), small indels (2-30bp), and single nucleotide polymorphisms (SNPs) (**Figure 2A-C** and **Supplementary Figure S6**). A total of 14,290 SVs were identified and further divided into 6 different categories: insertions, deletions, breakends (translocations), duplications, inversions, and inversed duplications (**Figure 2A**). Of these SVs, 86.8% were insertion and deletion variants, with 63.97% of the deletions and 50.35% of the insertions being larger than 100bp (**Supplementary Figure S7**). We analyzed the 663 deletions containing genes and identified 1,756 hemizygous genes (**Supplementary Table S10**), comprising about 4.9% of the *V. labrusca* gene models (**Figure 2D**, **Supplementary Figure S8**). We found ~48.4% (823) of the hemizygous genes had predicted biological functions, based on homology. Furthermore, the majority (99.2%) of hemizygous genes belonged to multi-copy gene families, while 14 hemizygous genes were identified as single copies with predicted fundamental functions, such as translation and DNA repair (**Supplementary Table S11**).

We further characterized the heterozygous genes in the *V. labrusca* genome at the gene (nucleotide variants between alleles) and protein (amino acid variants between alleles) levels by identifying SVs (31-859,735 bp), small indels (2-30 bps), and SNPs (**Figure 2E** and Methods). By using the SV insertions/deletions identified above, we detected 2,919 and 690 heterozygous genes at the gene and protein levels, respectively (**Supplementary Table S12**). Also, we identified 4,544 and 756 heterozygous genes on the gene and protein levels, resulting from small indel insertion or deletions in the *V. labrusca* genome (**Supplementary Table S13**). We identified 605,809 SNPs (representing 1 SNP every 828 bases) between the *V. labrusca* homologous chromosome sequences (Vlab_PG and Vlab_AG), with 40.2% of the total SNPs located within genic regions and 8.6% in exons (**Figure 2C**). Of the 35,915 *V. labrusca* gene models, 16,133 genes had anywhere from 1 to 335 SNPs in the gene body and 11,359 had SNPs in exons (**Supplementary Figure S9**). Further functional annotation identified 27,868 SNPs that would result in missense effects (protein sequence differences) in 8,693 heterozygous genes (**Supplementary Table S14**). Combining the results from the SVs, small indels, and SNP analyses, we classified the entire *V. labrusca* gene set into 1,756 hemizygous, 8,923 protein-level heterozygous, and 25,236 homozygous genes, demonstrating a substantial amount of intragenomic variations in this wild grapevine genome (**Figure 2E**).

Next, we investigated zygosity variation between a representative wild and cultivated genome, which originated from different breeding histories (natural versus selective breeding). We tested whether each collinear gene locus between the *V. labrusca* and available Chardonnay phased genome shared the same gene zygosity, by using the same pipeline as above to characterize 4,137 hemizygous, 12,420 protein-level heterozygous, and 17,901 homozygous genes in the Chardonnay genome, then established gene collinearity between these two genomes (**Figure 2E** and **Supplementary Table S15-S17**). Of the 1,756 hemizygous genes in *V. labrusca*, 95.8% were hemizygous only in *V. labrusca*, including 1,154 non-collinear hemizygous genes (no collinear genes found in Chardonnay) and 529 genes with different zygosity (either heterozygous or homozygous genes in Chardonnay) (**Figure 2F**). Similarly, of the 4,137 hemizygous genes in Chardonnay, 98.2% were Chardonnay-specific hemizygous genes (**Figure 2F**). A similar pattern was also observed for heterozygous and homozygous genes. Of the 8,923 heterozygous and 25,236 homozygous genes in *V. labrusca*, only 36.2% (3233/8923) and 27.7% (7026/25236) shared the same zygosity in Chardonnay (**Figure 2F**). Taken together, approximately half of the total collinear gene loci between *V. labrusca* and Chardonnay showed different states of zygosity, considering that only about 65% of the genes are collinear between the genomes.

### 3.3 Hemizygous genes originated from different gene gain-and-loss events in grapevine genomes

**Figure 3:**
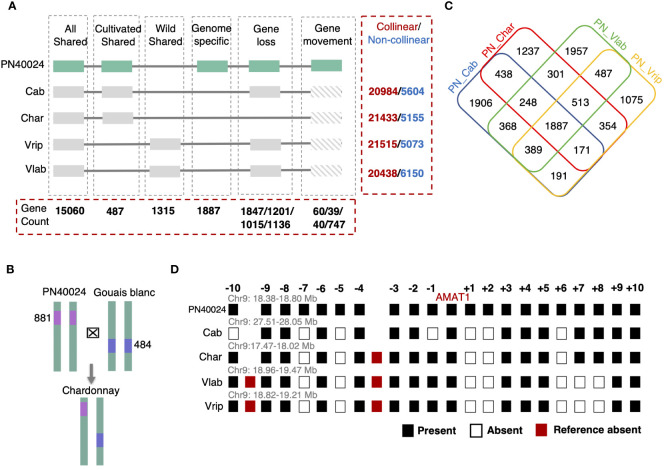
Collinear gene analysis among the five grapevine genomes. **(A)** Gene collinearity established using PN40024 as a reference. All genes were classified into four different subgroups: Genes shared by all the grapevine genomes (All Shared), genes found in just the cultivated genomes (Cultivated Shared), gene only present in wild grapevine genomes (Wild Shared), and genes only found in PN40024, with no collinear genes in other 4 species (Genome specific). In addition, gene loss and gene movement (translocation) events were identified in the Cabernet Sauvignon (Cab), Chardonnay (Char), *V. riparia* (Vrip), and *V. labrusca* (Vlab) genomes and the gene counts for these events are listed in this order in the figure, separated by slashes. The total number of collinear and non-collinear genes from pair-wise analysis with reference PN40024 are listed in the red dashed box to the right for the four other grapevine genomes. **(B)** The potential origination of hemizygous genes in Chardonnay with two putative parental lines. 881 PN40024 and 484 Gouais blanc non-collinear genes (absent from one of the two parental genomes) were identified as hemizygous genes in hybrid Chardonnay’s genome. **(C)** Non-collinear genes among grapevine genomes and the overlap detection. 1,887 genes are absent from all four genomes compared with PN40024. **(D)** Gene presence/absence patterns among the five grapevine genomes in the genomic region surrounding AMAT1 (10 genes upstream and downstream) on chromosome 9 highlights different gene gain-and-loss patterns among five grape genomes. Genome-specific genes are only displayed for PN40024.

We asked if the grapevine hemizygous genes could have arisen via the combination of parental genomes that had many non-collinear gene loci across chromosomes. First, we tested the abundance of non-collinear genes between five grapevine genomes: *V. labrusca*, *V. riparia*, PN40024, Cabernet Sauvignon, and Chardonnay. Many genes (20% to 39% of the total genes) were found to be non-collinear between any two of the grapevine genomes, even between different *V. vinifera* varieties (**Figure 3A** and **Supplementary Table S18**). Second, we tested whether these non-collinear genes could become hemizygous genes in hybrids. Theoretically, if a hemizygous gene in a hybrid originated from its parents, this gene should be present at that gene locus in only one of the two parents. Thus, we conducted an analysis with the Chardonnay genome and representative putative parental genomes, *V. vinifera* Gouais blanc and Pinot Noir (represented by PN40024) (Roach et al., 2018a). PN40024 was found to be the result of several rounds of selfing with cv. Helfensteiner, a cultivar that originated from crossing cv. Pinot Noir and cv. Schiava grossa (Velt et al., 2023). Though it is not a direct parent of Chardonnay, PN40024 shares some genetic background with Pinot Noir and can still be used to test the inheritance of hemizygous genes. After comparing the presence and absence of each of the 4,137 Chardonnay hemizygous gene within this trio-pedigree, we found that 881 hemizygous genes in the Chardonnay genome may have been inherited from Pinot Noir and 484 from Gouais blanc (Methods and **Figure 3B**), establishing a connection between hemizygous genes in progeny genomes and non-collinear genes in parental genomes. For hemizygous genes identified in the *V. labrusca* genome, we propose a similar explanation of inheritance of noncollinear genes from the parental plant genomes, which can be verified through future population genomics studies of the original dioecious *V. labrusca* population from which the Grem-4 accession was collected.

Non-collinear genes, like those in the Pinot Noir and Gouais blanc genomes that were subsequently passed on as hemizygous genes in Chardonnay, previously arose through gene gain-and-loss events somewhere along these grapevines’ lineages. By analyzing the noncollinear genes between PN40024 and the other four genomes, we found that the different genomes had unique gene gain-and-loss patterns (**Figure 3C**). For example, we comparatively analyzed genomic regions harboring the anthraniloyl-CoA:methanol acyltransferase (AMAT1) gene, which is responsible for the foxy flavor of some grape berries, and observed the different genomes had both shared and specific gene gain-and-loss events, which further demonstrates how gene gain-and-loss events can differentiate regions of the genome (**Figure 3D**). Established gene collinearity further led us to identify 15,060 All Shared collinear genes, 487 Eurasian Shared, 1,315 Wild Shared, and 1,887 genome-specific genes within the PN40024 reference genome (**Figure 3A**). We discovered over 79% of the genome-specific genes may have originated from gene duplications, based on homology. On the other hand, we found that gene loss and gene translocation were other major mechanisms to generate non-collinear genes (Methods and **Figure 3A**). Taken together, we propose that different gene gain-and-loss events in each grapevine lineage resulted in the large number of non-collinear genes and contributed to the overall gene content differences among the different grapevine genomes studied.

We next investigated the expression of collinear and non-collinear genes in PN40024, Cabernet Sauvignon, Chardonnay, and *V. labrusca* berry tissues and found collinear genes, on average, had significantly higher expression levels than non-collinear genes for all four grapevines (**Supplementary Figure S10**). We further compared the collinear gene expression in the berry tissue of *V. labrusca* and the three *V. vinifera* accessions and found thousands of collinear genes showed significant differential expression, including 2,250 differentially expressed genes between *V. labrusca* and all three cultivated grapevines (1,128 overexpressed and 1,122 underexpressed transcripts in *V. labrusca*) (**Supplementary Figure S11**). This result suggests, despite gene collinearity being conserved across different grapevine genomes, the expression of these genes can be differentially regulated, providing an extra layer of genetic diversity.

### 3.4 Segmental duplication is a key driving force for grapevine genome diversification

Segmental duplication (SD) has been suggested to highly impact genome evolution and species adaptation by creating genetic material (duplicated genes) that escapes ancestral selective constraints (Bailey et al., 2002; Dennis and Eichler, 2016). To understand whether SD plays a vital role in grapevine genome diversification, we conducted pairwise inter-chromosomal alignments to identify genome wide SDs (Methods). In each of the five grapevine genomes, we found 37,208 (Vlab) to 53,430 (Cab) recently originated SDs (>92% sequence identity), accounting for approximately 1/5 of the entire genome (**Supplementary Table S19-S23**). For each genome, 2.1-8.8% of SDs contained genes, which led to the discovery of 857 (PN40024), 1,981 (Cab), 3,349 (Char), 3,049 (Vlab) and 2,603 (Vrip) segmentally duplicated genes. Based on the previously identified gene collinearity, approximately 43.5-65.3% of SD-derived genes were found to be genome-specific, representing newly evolved genes in each grapevine genome (**Supplementary Table S24**). Of the genic regions that were segmentally duplicated multiple times, between 68.5 and 78.8% had only one SD that maintained a gene copy, suggesting most duplicated genes were subsequently disrupted and eventually lost after duplication (**Supplementary Figure S12**). For the remaining 30% of the SDs, which maintained multiple gene copies, we calculated the duplicated genes’ Ka/Ks ratios and identified 13-21% of duplicated genes were likely under positive selection (Ka/Ks>1) (**Supplementary Table S25**). These results indicate that these duplicated genes may provide a fitness advantage, possibly due to increased dosage, sub-functionalization, or neo-functionalization.

**Figure 4:**
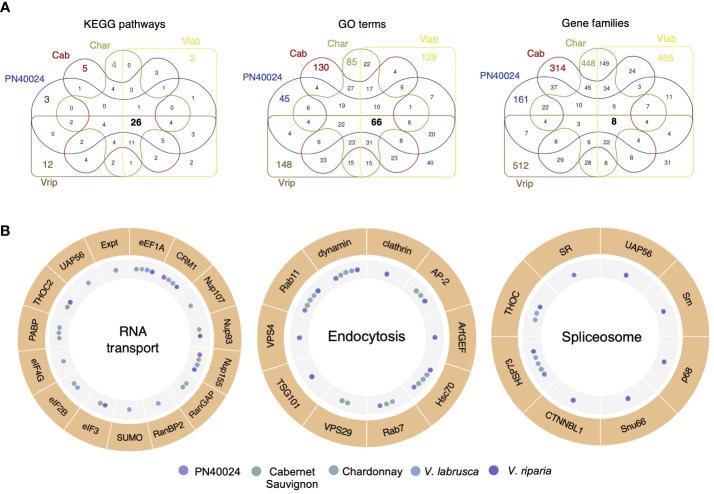
Segmental duplications caused amplification of different gene families among the five grapevine genomes. **(A)** Functional annotation of recently duplicated genome-specific genes. Venn diagrams of the number of genome-specific genes sharing KEGG pathways, GO terms, and gene families across the five grapevine genomes. **(B)** Differential gene duplication enrichment in shared pathways. RNA transport, Endocytosis, and Spliceosome pathways are shown as examples. The outer circle represents the genes in the pathways with duplicated genes, and the inner circle summarizes which genomes have duplicated genes in the corresponding steps of the pathway. A more detailed analysis can be found in **Supplementary Figures S14 and S15**.

To investigate the functions of the genome-specific genes which have been amplified and maintained within individual grapevine genomes, we first used the Kyoto Encyclopedia of Genes and Genomes (KEGG) to conduct pathway analyses. For each grapevine genome, 53 (PN40024), 63 (Cab), 73 (Char), 63 (Vlab), and 84 (Vrip) pathways were identified to be impacted by SD genes. Of them, 26 KEGG pathways were shared across the five grapevine genomes (**Figure 4A**). We continued to perform gene ontology (GO) analyses of the SD genes and identified 234 (PN40024), 429 (Cab), 369 (Char), 405 (Vlab) and 464 (Vrip) GO terms, with only 66 GO terms shared by all genomes (**Figure 4A**). Therefore, a considerable number of SD genes had GO terms that were genome specific or shared among a subset of the grapevine genomes (**Supplementary Figure S13**). For example, the Cabernet Sauvignon and Chardonnay genomes had an enrichment of SD genes involved in transmembrane transport and PN40024, Cabernet Sauvignon, Chardonnay, and *V. riparia* genomes had an enrichment of SD genes involved in RNA modification. Each genome had SD genes enriched in unique GO terms, as well. The *V. labrusca* genome had an enrichment of SD genes involved in DNA integration and wax biosynthesis, the *V. riparia* genome had an enrichment of flower development and stamen development SD genes, and the Chardonnay genome had an enrichment of sucrose metabolism and proteolysis SD genes, to highlight a few. This result suggests that genome-specific duplicated genes may have different roles in common biological pathways. Next, we narrowed the functional analysis to the gene family level. We assigned the duplicated genes to 302 (PN40024), 564 (Cab), 807 (Char), 781 (Vlab) and 686 (Vrip) gene families, and only eight gene families were shared by all genomes. This result clearly demonstrates that different grapevine gene families were amplified and maintained across the grapevine genomes, yet the SD genes maintained are enriched in the same metabolic pathways (**Figure 4A**).

Combining these results, we selected the 26 KEGG pathways shared by all genomes for further analysis. Even though the duplicated genes from different grapevine genomes were part of the same biological pathways, each genome varied in the number of genes amplified and maintained, as well as in the specific genes and their role in the biological pathways, across grapevine genomes (**Supplementary Figure S14**). For example, segmentally duplicated genes involved in the RNA transport pathway have been retained in all five grapevine genomes, but these genes differ in each genome, i.e., encode different enzymes in the pathway (**Figure 4B** and **Supplementary Figure S15**). Of them, *eEF1A*, *CRM1* and *Nup155* were duplicated in four of the five grapevine genomes, but *RanBP2*, *SUMO* and *UAP56* were only amplified and maintained in the *V. labrusca* genome. This phenomenon was common in other pathways as well, including Endocytosis and Spliceosome pathways (**Figure 4B**). In summary, we found that segmental duplications facilitated genome diversification through the interruption of genome collinearity and the creation of non-collinear duplicated genes.

### 3.5 Gene family evolution and species divergence

**Figure 5:**
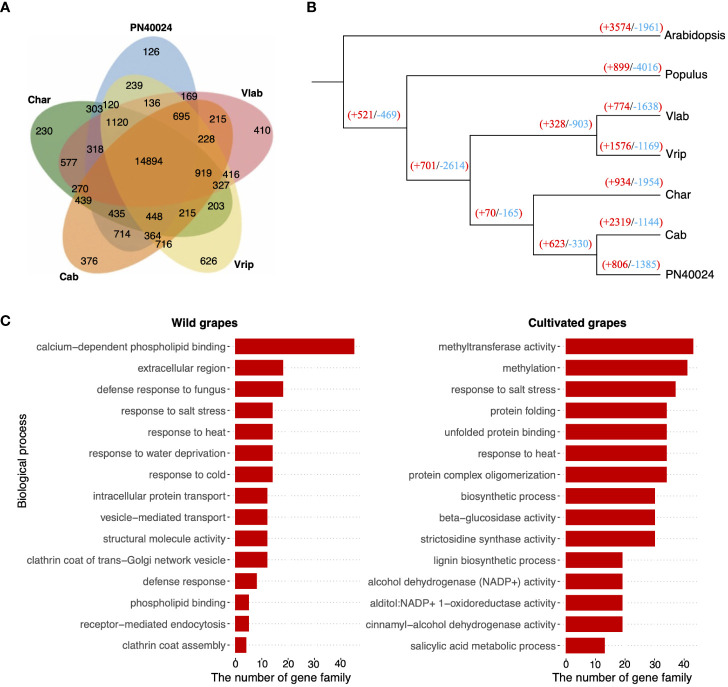
Gene family evolution. **(A)** Protein sequences from the 5 grapevine genomes were clustered into orthologous gene clusters and the relationship of the gene clusters across the 5 genomes is depicted in the Venn diagram. For example, 14,894 gene clusters were shared by all 5 genomes, but 410 gene clusters were only found in *V. labrusca* (Vlab). **(B)** Single copy orthologous genes were analyzed to produce a maximum likelihood species tree with Arabidopsis and Poplar as outgroups. The number of gene families that had undergone contraction and expansion are reported at each node: red numbers denote expanded gene families and blue numbers show contracted gene families. **(C)** Functional annotation of rapidly amplified gene families in wild (left) and cultivated (right) grapevines.

We compiled the 174,093 protein sequences across the five grapevine genomes and grouped them into gene clusters based on homology using OrthoFinder to further study grapevine gene family evolution. The gene clustering analysis identified 14,894 orthologous gene groups (containing a total of 101,178 genes), comprising 55.4% to 63.7% of the total genes across the five grapevine genomes. We detected 435 orthologous gene groups that were shared exclusively among the cultivated grapevines and 416 shared between the wild grapevines (**Figure 5A** and **Supplementary Table S26**) (In this analysis, as well as subsequent analyses, we categorized PN40024 as a cultivated grapevine, since it originated from cultivated parental varieties and served as a reference genome for cultivated grapevines). Functional annotation revealed that different gene ontology (GO) terms were enriched between cultivated and wild grapevines (**Supplementary Figure S16** and **Supplementary Table S27**). By counting the genome-specific genes, we estimated that 8 to13% of each grapevine genome consisted of unique gene content and another 2% were different between cultivated and wild species.

To better understand grapevine gene family evolution, we applied a “birth-and-death” model (De Bie et al., 2006) to test gene family contraction and expansion (**Figure 5B**) and identified 66 to 227 rapidly evolved gene families in each genome (**Supplementary Table S28**). A functional enrichment analysis revealed rapidly amplified gene families in both the cultivated and wild grapevine genomes were largely involved in environmental responses (**Figure 5C**). This result suggests gene amplifications involved in adaptation were under selection in both the cultivated and wild grapevine genomes. On the other hand, unique gene families were found to be amplified between them. For example, gene families involved in responses to cold were amplified in the two wild grapevine genomes, but contracted in cultivated grapevine varieties, suggesting these grapevine genomes were shaped, in part, by selective pressures of their local habitats (**Figure 5C** and **Supplementary Table S29–S32**). In addition, we noticed independent gene families with similar biological functions may experience opposite selection. For example, distinct gene families involved in heat and salt response were both amplified and contracted in cultivated grapevines (**Supplementary Table S31, S32**).

### 3.6 NLR gene family evolution among grapevine species

**Figure 6:**
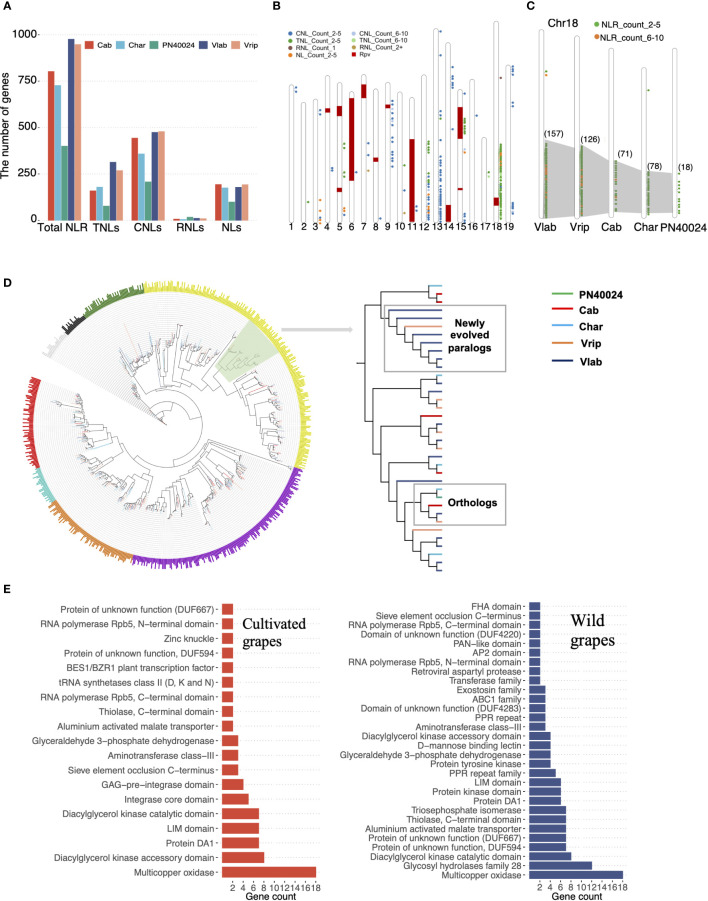
NLR gene family evolution. **(A)** NLR gene identification. Total NLRs were characterized in the five grapevine genomes and classified into four subgroups based on the combination of their conserved domains: TNL (TIR-NLR), CNL (CC-NLR), RNL (CC_R_-NLR) and NLs (NB-and-LRR-only). **(B)** Distribution of NLR genes and 12 RPV loci across 19 chromosomes in *V. labrusca*. Each colored dot represents the number of NLR gene copies with a specific conserved domain located in that region of the chromosome and the “Count” value specifies the number of gene copies. Previously identified RPV loci are denoted by red blocks. **(C)** The expansion of the NLR gene cluster on Chr18 across cultivated and wild grapevines. **(D)** Phylogenetic analysis of all TNLs in the five grapevine genomes. Large clades are depicted with different colors. Branch colors indicated different grapevine genomes. The clade highlighted in light green is zoomed in to show the duplication process in *V. labrusca*. **(E)** Integrated domain (ID) annotation between cultivated and wild grapevines (only showing IDs that appeared in more than 2 NLR genes).

North American wild grapevines have strong resistance to several major grapevine pathogens, including downy mildew and powdery mildew (Qiu et al., 2015; Divilov et al., 2018). Despite previous studies demonstrating a strong association between resistance to mildew pathogens and nucleotide-binding leucine-rich repeat receptor (NLR) genes, the genomic dynamics driving the evolution of NLR genes in grapevine is still not well understood (Feechan et al., 2013; Van de Weyer et al., 2019; Goyal et al., 2020). We characterized a total of 3,852 NLR genes in the five grapevine genomes, from 400 in PN40024 to 976 in *V. labrusca* (**Figure 6A** and **Supplementary Table S33**). The NLR gene count in PN40024 was consistent with previous studies (Goyal et al., 2020) but we detected a much higher number of NLR genes in the other two cultivated grapevine genomes, suggesting that previous results based on the PN40024 Sanger sequencing reference genome may have underestimated the NLR gene copy number. As expected, the NLR gene family expanded greatly in the wild grapevine genomes. The TNL subgroup underwent a major expansion, raising the ratio between TNL and non-TNL genes from 1:4 in the cultivated grapevine genomes (Yang et al., 2008) to 1:2 in the wild grapevine genomes (**Figure 6A**). NLR genes are often found in gene clusters and we found the expanded NLR gene clusters were located in regions of the genomes which were previously identified as *P. viticola* resistance (RPV) loci through genetic mapping (Maul, 2020) indicating some of these duplicated NLR genes may contribute to downy mildew resistance (**Figure 6B**).

We further focused on the TNL supercluster on chr18, accounting for 45.4% of the total TNLs, and observed that the TNL genes still maintained high collinearity among the five grapevine genomes, but an obvious expansion was found in the wild grapevine genomes: 18 TNL genes were present at this locus in PN40024 compared to 157 copies in *V. labrusca* (**Figure 6C**). Phylogenetic analysis revealed that grapevine TNLs are separated into seven clades (**Figure 6D**). Within each clade, TNL orthologs from the five grapevine genomes were clustered and some branches were formed with recently duplicated genes in the wild grapevines, suggesting the ancient gene copies were still maintained, but the additional duplicated TNL gene copies in both *V. labrusca* and *V. riparia* may have had a selective advantage (**Figure 6D**). In addition, we found that NLR genes in the wild grapevines had more specific integrated domains (IDs), which may reinforce effector detection ability for more diverse pathogens (**Figure 6E**) (Baggs et al., 2017; Grund et al., 2019). Taken together, these results suggested that TNL gene expansion and higher ID diversity may contribute to enhanced disease resistance in wild grapevines.

### 3.7 TE insertion polymorphisms differentially regulate collinear genes

Transposable elements (TEs) are known to play a key role in genome evolution and adaptation by interrupting gene structure, impacting expression profiles of nearby genes, or affecting recombination (Bennetzen and Wang, 2014; Hirsch and Springer, 2017). To explore how TEs shape the grapevine genomes, we investigated TE insertion polymorphism in 12,155 collinear genic regions, focusing on LTR retrotransposons and MITEs (**Supplementary Table S34-36**). The results showed that 1,192 and 4,078 collinear genes in all five grapevine genomes contained LTR and MITE insertions within the gene body, respectively (**Supplementary Figure S17,18**). Additionally, 2,215 and 2,047 of the collinear genes had TE insertions within 1 kb upstream and 1 kb downstream, respectively, suggesting TE insertion polymorphisms existed in around 61% of the total collinear genic regions. Within gene regulatory regions (1 kb upstream), 538 collinear genes contained TE insertions exclusively in the two wild grapevines and 489 collinear genes contained TE insertions exclusively in the three cultivated grapevines. Furthermore, hundreds of genome-specific TE insertions were also detected in individual genomes, implying these TE insertion polymorphisms may contribute to differential expression of these collinear genes across the grapevines.

**Figure 7:**
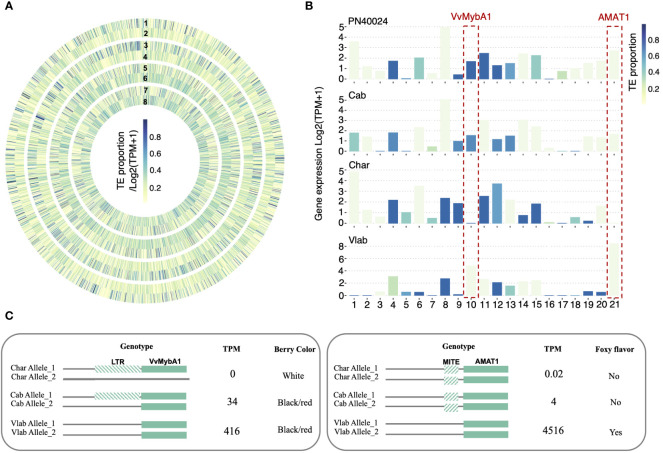
TE insertion polymorphism and gene expression regulation. **(A)** A heat map displaying TE insertion polymorphisms and the potential relationship between gene expression and TE proportion within the 1 kb upstream regions of collinear genes. Circles 1, 3, 5, and 7 represent the TE proportion and Circles 2, 4, 6, and 8 represent gene expression. Circles 1 and 2 correspond to PN40024, 3 and 4 to Cabernet Sauvignon, 5 and 6 to Cahrdonnay, and 7 and 8 to *V. labrusca*. The same color key corresponds to both the TE proportion and gene expression values, where the value intervals are linearly mapped to 9 continuous colors ranging between yellow and blue. **(B)** Bar graphs of 21 example genes in which the proportion of TE insertions inversely correlate to gene expression. Bar color indicates TE proportions, and the y-axis represents gene expression levels. **(C)** Two examples showing TE regulated gene expression, VvMybA1 and AMAT1. Upstream regions of genes were genotyped to identify homozygous/heterozygous TE insertions and were associated with gene expression levels (TPM) and corresponding phenotypes.

Our previous analyses revealed that collinear genes across different grapevine genomes can show differential expression (**Supplementary Figure S11**). To test whether TE insertion polymorphisms may cause gene differential expression, we further focused on the 1 kb upstream regulatory regions of the collinear genes. We identified 3,055 collinear genes showing TE-insertion polymorphism across the five grapevine genomes (i.e., at least one gene’s 1kb upstream region does not have TEs inserted and one gene’s 1kb upstream region consists of more than 20% TEs) (Methods). We found 892 genes (29.2%) followed the expected inverse correlation between TE proportion and gene expression (**Figure 7A, B**). We tested our TE polymorphism dataset with two previously reported grapevine genes that have expression regulated by TEs, VvMybA1 and AMAT1 (**Figure 7B**). VvMybA1 was reported to be a key gene in the anthocyanin synthesis pathway. One LTR/Gypsy insertion in the regulatory region of VvMybA1 was found to interrupt gene expression and cause the grape berry color to turn from red to white (Kobayashi et al., 2004). However, in Chardonnay, a white berry cultivar, previous studies also showed a large deletion occurred in one of the chr2 homologous chromosomes, which removed one of the functional VvMybA1 alleles, resulting in a hemizygous locus (Roach et al., 2018a; Zhou et al., 2019). In our dataset, we confirmed that the remaining VvMybA1 copy in the Chardonnay genome did have an LTR insertion in the regulatory region of this allele, which disrupts the expression of this gene (**Supplementary Figure 19A**). Within the *V. labrusca* genome, a red berry species, the VvMybA1 locus is homozygous for no TE insertion (**Supplementary Figure 19B**). Consistent with this genotype, the MybA1 gene was highly expressed in *V. labrusca*. In Cabernet Sauvignon (red berry), we confirmed that the MybA1 gene locus was heterozygous, with one uninterrupted functional copy and one TE-inserted silenced copy and observed moderate gene expression levels (**Figure 7B, C** and **Supplementary Figure 19C**). As another example, AMAT1 encodes an acyltransferase which catalyzes the formation of methyl anthranilate. This compound was proposed to be responsible for the ‘foxy’ flavor of the *V. labrusca* fruit and its hybrids (Wang and De Luca, 2005). We analyzed the TE insertion and gene expression of the AMAT1 gene and identified the MITE insertion in cultivated grapes, which was previously associated with reduced methyl anthranilate production (Yang et al., 2020). Consistently, we observed a 4,000-fold drop in the gene expression level of AMAT1 in cultivated grapes compared with *V. labrusca* (**Figure 7C**). Although the gene expression regulation is thought to be more complicated than solely being regulated by this TE insertion, our analysis demonstrated that TE insertion polymorphism is a potential mechanism for differential gene expression of collinear genes in grapevines, adding an additional layer of genetic diversity.

## 4 Discussion

In contrast to self-pollinating diploid species, which have two sets of homologous chromosomes with limited allelic variation, obligate outcrossing diploids, such as wild grapevines, acquire greater haplotype diversity, leading to higher intragenomic variation. Based on this, we expected the genomes of dioecious wild grapevines to have high intragenomic sequence variation. But our comprehensive analyses found that the intragenomic variation for the *V. labrusca* genome was only 4.99% and the *V. vinifera* ssp. *sylvesteris* genome was 7.83%, whereas the intragenomic sequence variation of cultivated Chardonnay and Cabernet Sauvignon genomes were about twice as high, at 10.08% and 16.98%, respectively. The increased heterozygosity in the cultivated grapevine genomes was likely due to the hybridization of distinct grapevine genotypes, followed by vegetative propagation, contributing to the increase and maintenance of heterozygosity in the genomes (Jaillon et al., 2007; Zhou et al., 2019). The lower intragenomic variation in the wild grapevine genomes studied, compared to the cultivated genomes, could be due to limited genotypic diversity present within their natural populations and small effective population size, but future analyses of these wild grapevine populations will need to be performed to provide insight into whether this hypothesis holds true. Multiple studies that have investigated the genetic diversity of wild and cultivated grapevine populations using molecular markers found the observed heterozygosity (Ho) of wild grapevines to be lower than the expected heterozygosity (He), or, in some cases close to what was expected, whereas the genetic diversity of cultivated grapevines was higher than the expected heterozygosity, providing support for this trend at the population level (De Andrés et al., 2012; Emanuelli et al., 2013; Riaz et al., 2018).

The intragenomic variation was not only distributed within the intergenic regions but impacted numerous genic regions, as well. About 30% of the gene loci in *V. labrusca*, and 48% in Chardonnay, were identified as heterozygous or hemizygous and about half (54.6%) of the total gene loci between the two had different zygosity. The observed differences in gene zygosity provide insights into past observations of inbreeding depression and hybrid vigor in grapevine breeding programs (Patel et al., 2018). Inbreeding can increase the accumulation of homozygous recessive alleles and result in the loss of hemizygous genes in one forth of the progeny. Clonal propagation maintains these inbreeding consequences and additional somatic mutations can also contribute to intergenomic variations between clones, leading to phenotypic variation, as observed in Chardonnay and Zinfandel clones (Roach et al., 2018a; Vondras et al., 2019). However, hybridization can increase the proportion of heterozygous genes and introduce new combinations of hemizygous genes in the progeny.

A study by Zhou et al. (2019) discovered over 5,000 hemizygous genes in the Chardonnay genome and found the heterozygosity of Chardonnay was 11% higher than its wild progenitor, *V. vinifera* ssp.*sylvestris*. Our results agree with the Zhou et al. (2019) and Roach et al. (2018a) findings of high hemizygosity in Chardonnay, as we discovered 12% (4,137) of the genes in the Chardonnay genome we studied were hemizygous (Roach et al., 2018a; Zhou et al., 2019). The parental contribution of the Pinot Noir and Gouais blanc genomes to their progeny Chardonnay genome was previously reported (Roach et al., 2018a), so we used this trio-pedigree to determine whether hemizygous genes in Chardonnay were inherited or if they were the result of gene loss events in the Chardonnay genome. We found that at least a third of the hemizygous genes resulted from the inheritance of non-collinear genes from the parent genomes. The use of the PN40024 genome in these analyses, instead of the actual Pinot Noir parental genome, likely contributed to the lower contribution of non-collinear genes detected, but our results still support that the inheritance of non-collinear genes from the parent genomes greatly contribute to creating hemizygous genes in the progeny.

Whether the presence of these hemizygous genes is beneficial is dependent on their function and effect on fitness or agricultural traits. The hemizygous genes identified in Chardonnay by Zhou et al. (2019) were enriched in defense response functions, and hemizygosity also affected berry color in Chardonnay, an important crop trait, which suggests hemizygous genes can contribute to desirable agronomic traits. On the other hand, in apple, another clonally propagated crop, a hemizygous somatic deletion, resulting in the loss of the *MdACT7* functional allele in Autumn Gala, contributed to a delay of fruit maturation (Ban et al., 2022). Fruit maturation time is an important trait and early maturing cultivars may be more desirable for some orchards. Hemizygosity can lead to the exposure of deleterious recessive alleles that would otherwise be masked by its homologous gene pair, which could have negative consequences, as seen in apple. Subsequent clonal propagation would maintain these deleterious mutations in the genome (Allaby, 2019).

We discovered 1,756 hemizygous genes in the *V. labrusca* genome, less than half the number of hemizygous genes found in the Chardonnay genome, providing context for gene hemizygosity in wild grapevine genomes. The majority of the hemizygous genes found in the *V. labrusca* genome were members of multigene families, which may mean there is some level of functional redundancy, decreasing the impact of losing these hemizygous genes in part of the of progeny. About 14% of the hemizygous genes in the *V. labrusca* genome were identified to be single copy genes and predicted to be involved in fundamental functions, such as DNA repair and translation, which could have a more impactful effect if these single copy hemizygous genes are lost in half the progeny via sexual reproduction. Understanding gene collinearity in grapevine genomes, as well as the functions of hemizygous genes, can help inform grapevine breeding efforts. This concept can also be extended to other vegetatively propagated perennial plants, though further investigation of the presence of hemizygous genes in these plant genomes is needed to determine their impact on phenotypes.

Understanding genome diversification provides fundamental knowledge for explaining the association between genetic and phenotypic variations, species divergence, and local adaptation through an evolutionary lens (Savolainen et al., 2013). Comparative analyses between the five grapevine genomes revealed ~1/3 of the total gene models were not found at collinear positions between cultivated and wild grapevine genomes. Of course, the extent of collinearity detected in our study could be impacted by genome assembly strategy and assembly quality. For example, a new telomere-to-telomere reference genome for PN40024 was released after completion of this project, which has substantial improvements compared with previous version (Shi et al., 2023) and could be used to further validate the grapevine genome assemblies and gene collinearity across genomes. Even so, a study that haplotyped the *Vitis* collinear core genome reported only 37~54% of grapevine genes were collinear across genomes (Zou et al., 2020), supporting our findings of a high number of non-collinear genes across grapevine genomes and suggesting different gene gain-and-loss events have occurred at different loci since divergence from a common ancestor. This trend is not unique to grapevine; a survey of the structural variation of 3,000 rice genomes found significant variation in the number of “deleted genes” across rice varieties (Fuentes et al., 2019) and a comparison between the B73 and Mo17 maize lines revealed about 10% of the annotated genes were non-syntenic (Sun et al., 2018), demonstrating that difference in gene presence and absence significantly contributes to genome diversification of other crop varieties, as well.

TEs also significantly contribute to genome diversification and LTRs, specifically, are a main contributor to variation in plant genome sizes (Liu et al., 2020). We found that the LTRs composed 23.4-32.7% of the grapevine genomes studied, which is less than some other perennial crops, such as Chinese plum (*Prunus salicina* Lindl.) and wild pear (*Pyrus betuleafolia*), with LTRs comprising 39.8% and 33.1% of the genomes, respectively (Huang et al., 2021; Dong et al., 2020). Furthermore, TEs can affect gene transcription; TE polymorphisms were detected in 61% of the collinear genic regions across the 5 grapevine genomes, and more than 3,000 collinear genes had TE insertion polymorphisms in the 1kb upstream regulatory region, which correlated with expression differences for hundreds of these genes. TEs have been demonstrated to play key roles in genome structure variation, adaptation, and speciation (Serrato-Capuchina and Matute, 2018; Schrader and Schmitz, 2019; Mérot et al., 2020). For example, a Ty1/copia-like retrotransposon disrupted the *GmphyA2* gene in soybean and resulted in photoperiod insensitivity, which allowed for adaptation to higher latitudes (Liu et al., 2008; Kanazawa et al., 2009). Although TEs in the regulatory regions of MybA1 and AMAT1 are present in cultivated grapevines, reducing anthocyanin and methyl anthranilate levels, respectively, our analyses of *V. labrusca* confirmed the absence of TEs in the regulatory regions of MybA1 and AMAT1, allowing for production of these compounds in *V. labrusca* berries (Hirsch and Springer, 2017; Uzunović et al., 2019). Variation in TE insertions contribute to grapevine genome diversification.

Segmental duplications drive genome evolution by creating new genetic material, leading to genetic novelty (Dennis and Eichler, 2016). A past study of the PN40024 genome estimated 17.47% of the grapevine genome was comprised of SDs (Giannuzzi et al., 2011) and our comparative analysis concurred, revealing about 16.6-24.8% the grapevine genomes studied consisted of recent SDs (**Supplementary Table S22-S26**). We also found 2.1%-8.8% of the total genes in the grapevine genomes arose recently through SDs and 50% of these SD-derived genes were only found in one of the five genomes (genome-specific), providing support that SD is an important mechanism for the evolution of genetic novelty in these grapevine genomes. Our study further demonstrated that SD is an important mechanism for specialized gene family expansion in grapevine genomes, even though the recently duplicated, genome-specific genes may still be under purifying selection. Gene families involved with biotic and abiotic stress responses, which are central to local adaptation, were amplified in both cultivated and wild grapevines, but gene families involved in response to specific stressors were rapidly amplified in different species. For example, the wild grapevines included in this study were adapted to withstand below-freezing winter temperatures and drought conditions of the North Eastern United States (Reynolds and Reisch, 2015). Studies have also demonstrated *V. labrusca* and *V. riparia* can have superior resistance to fungal pathogens compared to *V. vinifera* (Cadle-Davidson, 2008; Cadle-Davidson et al., 2011). We found gene families involved in defense response to fungus, water deprivation, and cold were considerably expanded in the wild grapevine genomes we investigated, but not in the cultivated grapevine genomes, whereas gene families involved in response to heat and salt stress were amplified in both. Recent studies provide additional support for expansion and differential expression of gene families involved in abiotic and biotic stress tolerance in wild grapevine (Patel et al., 2020; Cochetel et al., 2021; Wang et al., 2021). These results suggest gene families involved in abiotic and biotic responses expanded and diversified in the wild and cultivated grapevine genomes we studied, but each genome appears to have maintained genes that could provide an adaptive fitness advantage for their local environment.

SDs have been identified as contributing important adaptive traits in other crop species, as well. A tandemly duplicated ethylene response factor gene, *Sub1A*, was discovered to provide submergence tolerance in *O. sativa* ssp. indica FR13A and copy number variation of specific C-repeat binding factor (CBF) genes, specifically *HvCBF4* and *HvCBF2*, provide increased tolerance to frost in barley (Xu et al., 2006; Francia et al., 2016), demonstrating SDs are important sources of genes conferring tolerance to abiotic stress. SDs can also create genetic variation that contributes to important crop domestication traits. For example, a gene duplication of the fw3.2 locus in tomatoes, resulted in two identical copies of *SIKLUH* genes, doubling the expression of the gene, which was identified as contributing to larger fruit size and greater fruit weight, a desirable domestication trait (Alonge et al., 2020). In this case, the duplicated gene didn’t evolve a unique function but the additional gene copy with a redundant function altered the transcript dosage, contributing to changes in phenotype (Lye and Purugganan, 2019).

The NLR gene family is an excellent example of fitness-related gene family expansion. Resistance genes, such as NBS-LRR genes, protect plants from pathogens and past studies have reported that duplications can impact the evolution of NBS-LRR disease resistance genes (Leister, 2004). For example, tandem duplication was a major driving force behind the expansion of NBS-LRR gene clusters in white Guinea yam (*Dioscorea rotundata*), wild pear (*Pyrus betuleafoilia*), and in the legume family (Zhang et al., 2020; Dong et al., 2020; Shao et al., 2014). We observed an expansion of 145 NBS-LRR genes in *V. riparia* (947 total NBS-LRR genes) and 174 in *V. labrusca* (976 total NBS-LRR genes) compared to Cabernet Sauvignon (802 NBS-LRR genes), which had the highest number of NBS-LRR genes of the three cultivated varieties we studied. An expansion of 145 NBS-LRR genes was also reported in *Muscadinia rotundifolia* compared to Cabernet Sauvignon (Cochetel et al., 2021). In Oryza, the opposite trend was observed in an investigation of 13 domesticated and wild rice genomes. NLR gene copy number was higher in domesticated *O. sativa* ssp. indica and *O. sativa* ssp. japonica compared to wild *Oryza* species, presumably due to breeding for increased NLR diversity (Stein et al., 2018). When comparing total number of NLR genes across recently studied wild and crop plants, we see that grapevine has considerably more NLR genes than rice (237-535 NLR genes; Stein et al., 2018), white guinea yam (167 NLR genes; Zhang et al., 2020);, Tomato (264-332 NLR genes; Seong et al., 2020), wild pear (573 NBS genes; Dong et al., 2020), and soybean (Glycine max (L.) Merr.; 503 NLR genes; Kim et al., 2021). PN40024 only had 400 NLR genes, but we discovered 727 and 802 in Chardonnay and Cabernet Sauvignon, and 947 and 976 in *V. riparia* and *V. labrusca*, respectively. Our phylogenetic analysis of the TNLs revealed recent duplications in wild grapevine lineages contribute to the increased number of NLR genes in the wild grapevine genomes studied. Foria et al. (2020) reported that the genes conferring resistance to *Plasmopara viticola* (downy mildew) in the resistant wild grapevine haplotype, Rpv3-1, were a pair of tandemly duplicated TNL genes found on the lower arm of chromosome 18. We found that the TNL subgroup of NLR genes underwent a major expansion on the lower arm of chromosome 18 in the wild grapevine genomes we studied, with 141 TNL copies in this region of *V. labrusca* alone, many of which arose through recent duplications. Interestingly, different wild grapevine species, and accessions within species, have shown variation in resistance to different pathogen isolates, which leads to the hypothesis that the considerable expansion of the NLR genes observed in the wild grapevine genomes may reflect an adaptive response to strong selective pressures exerted by the arms race between virulence of local pathogens, such as downy mildew, and resistance to these pathogens in grapevines, which is increased and/or diversified through gene duplication and specialization, providing an adaptive advantage (Gadoury and Pearson, 1991; Cadle-Davidson et al., 2011). Our study generated a set of NLR candidate genes that may confer broad or enhanced resistance in wild grapevine species; Additional functional studies of these genes will identify gene targets for future introgression through molecular breeding or genetic editing to increase resistance in cultivated varieties. Further comparative analyses between “New World” wild grapevine species and cultivated Eurasian grapevines will identify additional genetic variation to target for crop improvement and sustainable viticulture.

## Data availability statement

The datasets presented in this study can be found in online repositories. The names of the repository/repositories and accession number(s) can be found below: https://www.ncbi.nlm.nih.gov/, PRJNA701071. The V. labrusca genome assembly and annotation can be found at https://figshare.com/projects/Vitis_labrusca_sequencing_project/101669 and custom codes used for data analyses are available on GitHub (https://github.com/BoLi-OSU-PlantGenome/Vitis_labrusca_project).

## Author contributions

AG and BL conceptualized the project. BL performed the genomic analyses. BL and AG analyzed the data. BL and AG wrote and edited the manuscript. All authors contributed to the article and approved the submitted version.
