# Supplementary material for: Vitis labrusca genome assembly reveals diversification between wild and cultivated grapevine genomes
Source: Front Plant Sci. 2023 Aug 31;14:1234130. doi: 10.3389/fpls.2023.1234130 (PMC10501149; doi:10.3389/fpls.2023.1234130)
Supplement: Supplementary file 1 [file DataSheet_1.pdf]

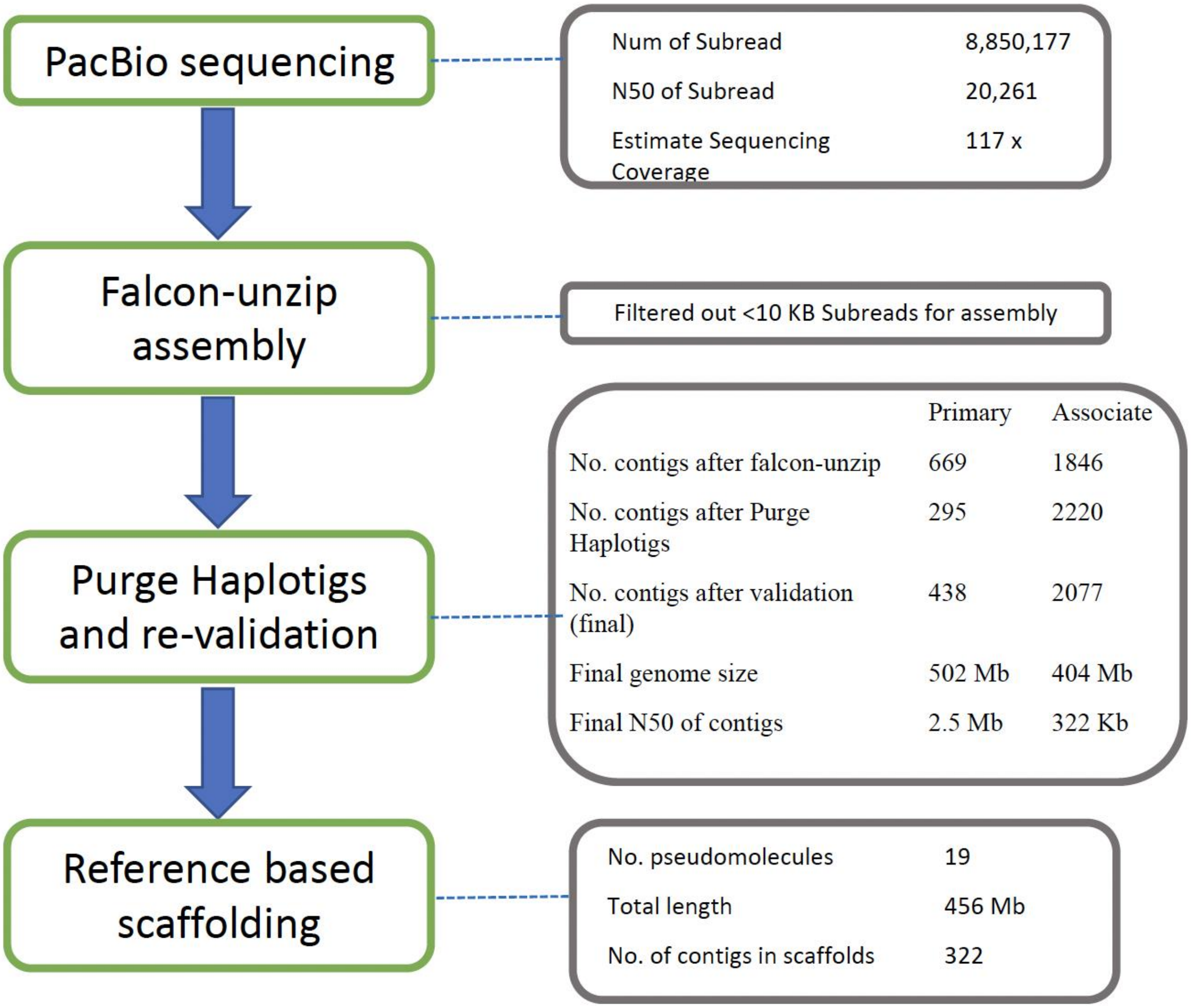

Supplementary Figure S1. *Vitis labrusca* genome sequencing and assembly pipeline. The three major steps of *V. labrusca* genome assembly are listed in the green boxes and the statistic summaries for each step are listed in the beige boxes. The details about the genome assembly strategy can be seen in the Methods.

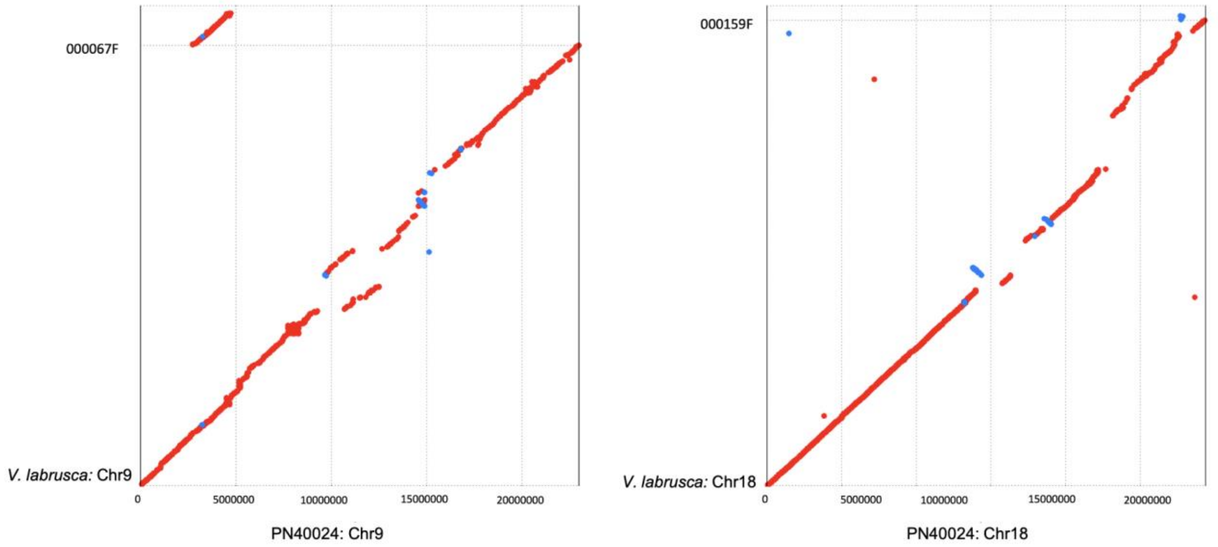

Supplementary Figure S2. Reference-based validation of contigs identified by Purge Haplotigs tool. To validate whether the contig identified by Purge Haplotigs should be removed from the p-group to the a-group, we aligned this contig and other p-contigs onto the corresponding chromosomes from the *V. vinifera* reference genome (PN40024). If there was an overlap (000067F), this was further support for reassigning this contig to the a-contig group; if there was no overlap (000159F), we kept this contig within the p-group for further analysis. Red dots represent direct alignments; blue dots represent inversed alignments.

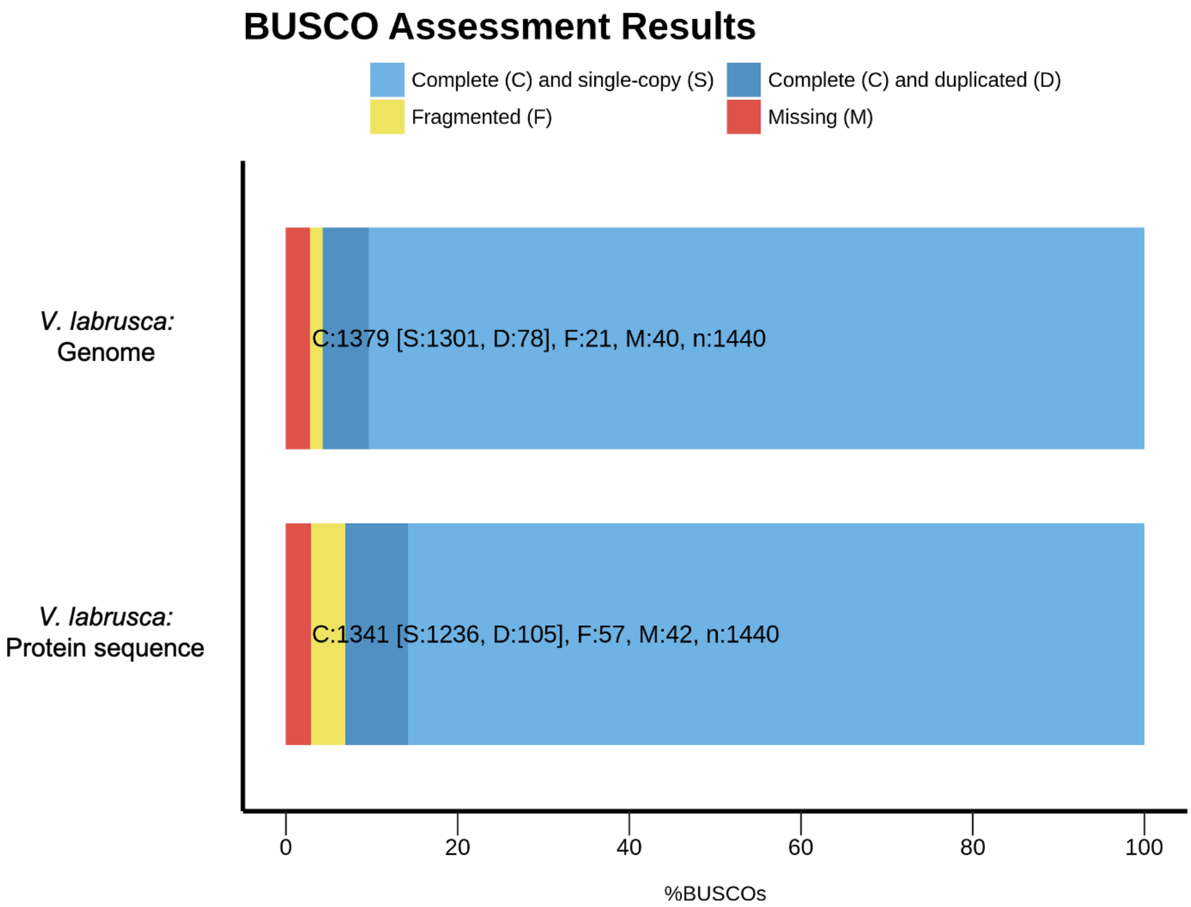

Supplementary Figure S3. BUSCO assessment of genome assembly (Vlabrusca_Genome) and gene annotation (Vlabrusca_protein) of *V. labrusca*. In 1,440 BUSCO gene models, both genome sequences and gene annotation results showed high coverage and very few missing gene models.

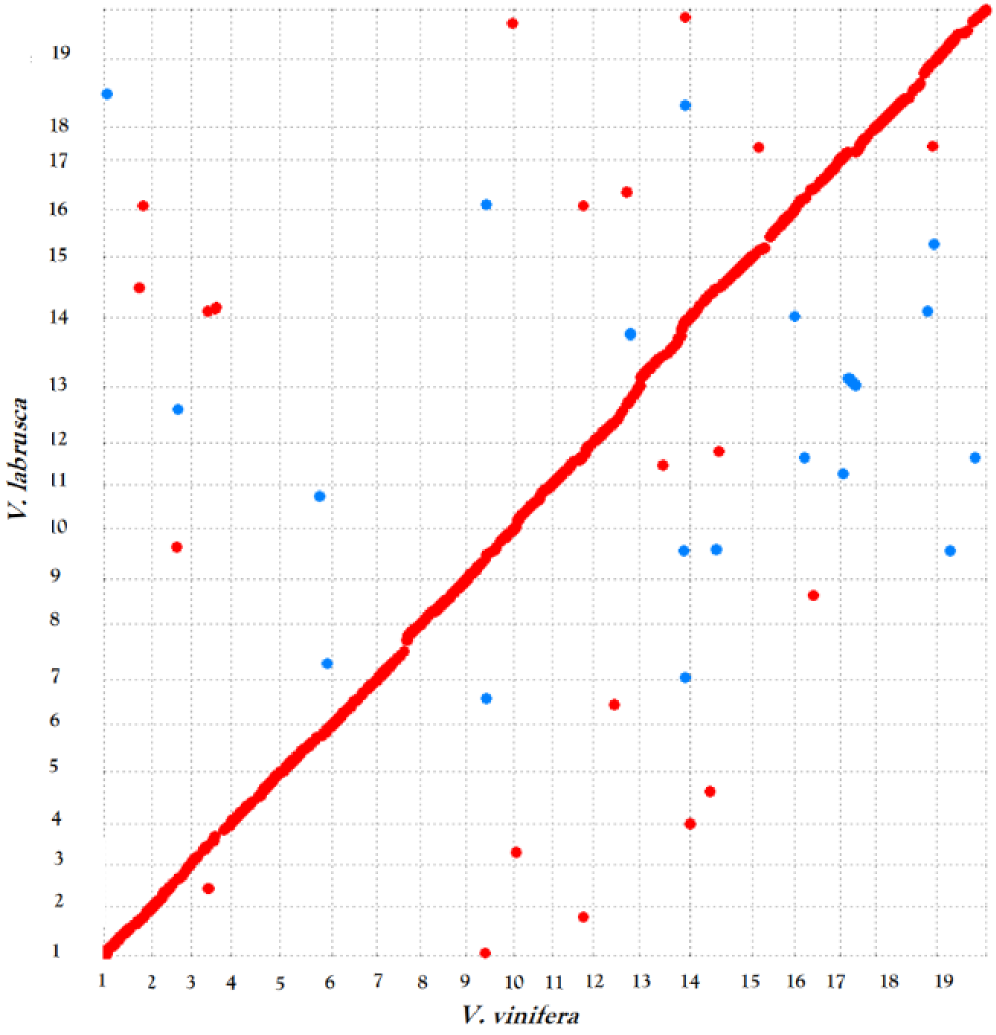

Supplementary Figure S4. Dot-plot between the sequences from 19 pseudomolecules from both *V. vinifera* (PN40024) and *V. labrusca* demonstrated overall high sequence collinearity. Red dots represent direct alignments and blue dots represent inversed alignments.

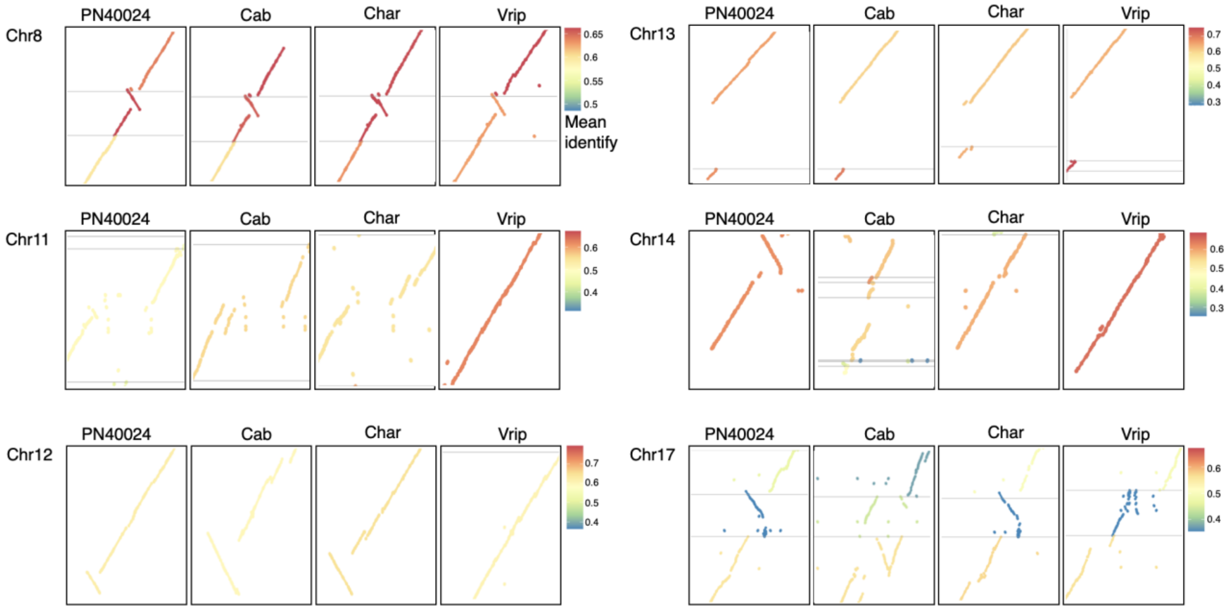

Supplementary Figure S5. Validation of large SVs between *V. labrusca* and *V. vinifera* PN40024 on six chromosomes. The SVs detected between *V. labrusca* and PN40024 can be further validated by two other *V. vinifera* genomes, Cabernet Sauvignon (Cab) and Chardonnay (Char), and *V. riparia* (Vrip). The results supported that some SVs identified between *V. labrusca* and *V. vinifera* are likely true (Chr11, Chr12 and Chr17), some SVs may be *V. labrusca* specific or assembly errors (Chr8 and Chr13), some may be PN40024 specific or assembly errors (Chr14).

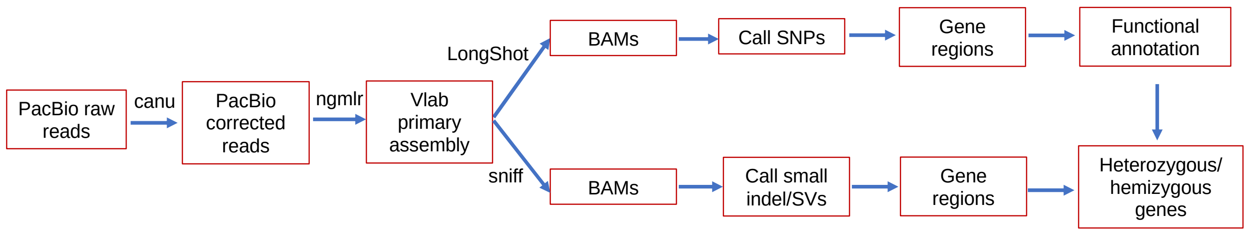

Supplementary Figure S6. Research pipeline to uncover three different categories of genetic variations, including structural variations, small indels, and SNPs, and to identify heterozygous or hemizygous genes between two subgenomes in *V. labrusca*.

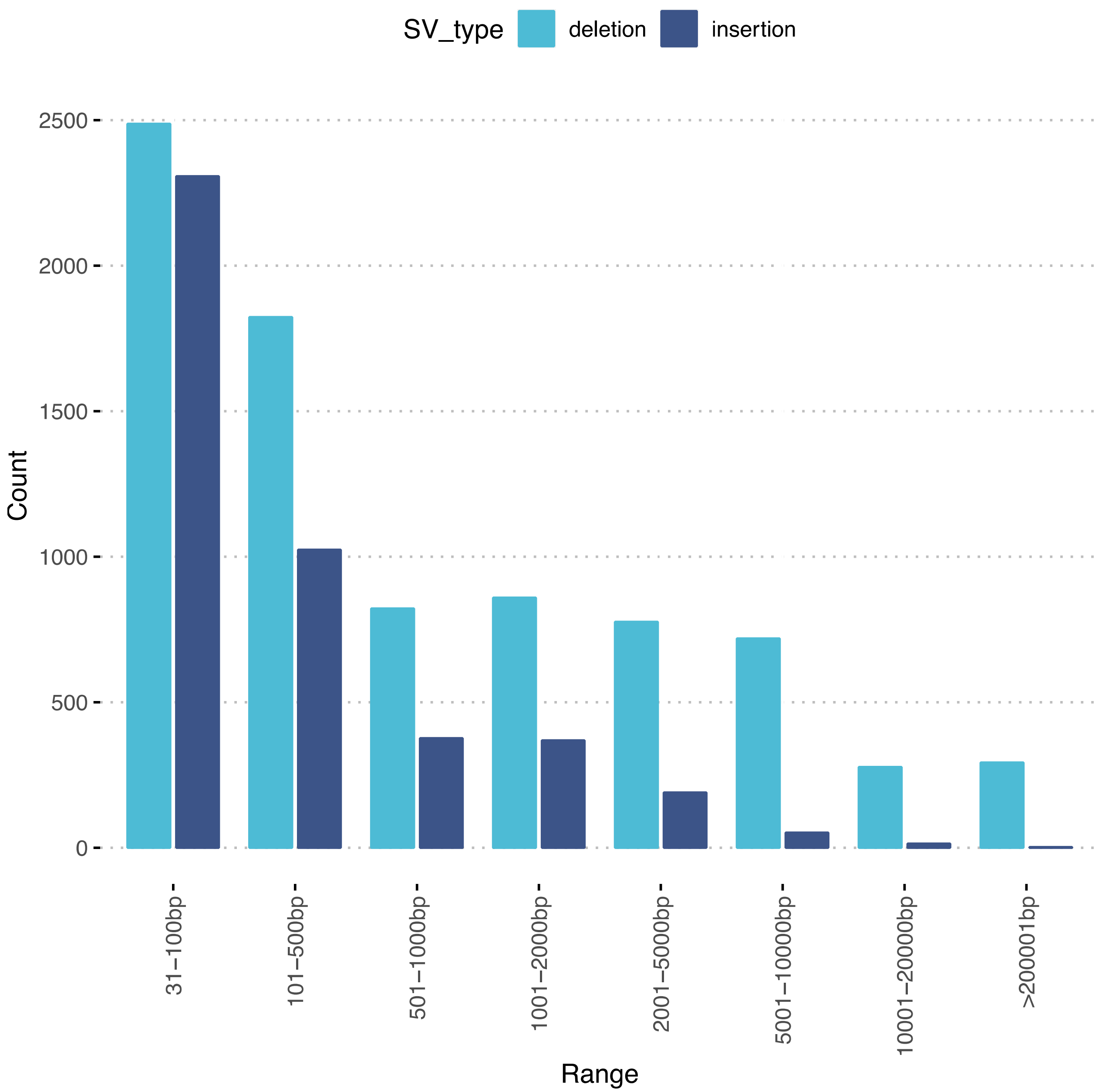

Supplementary Figure S7. Sequence length of insertions and deletions, which are the most prevalent structural variations discovered in the *V. labrusca* genome.

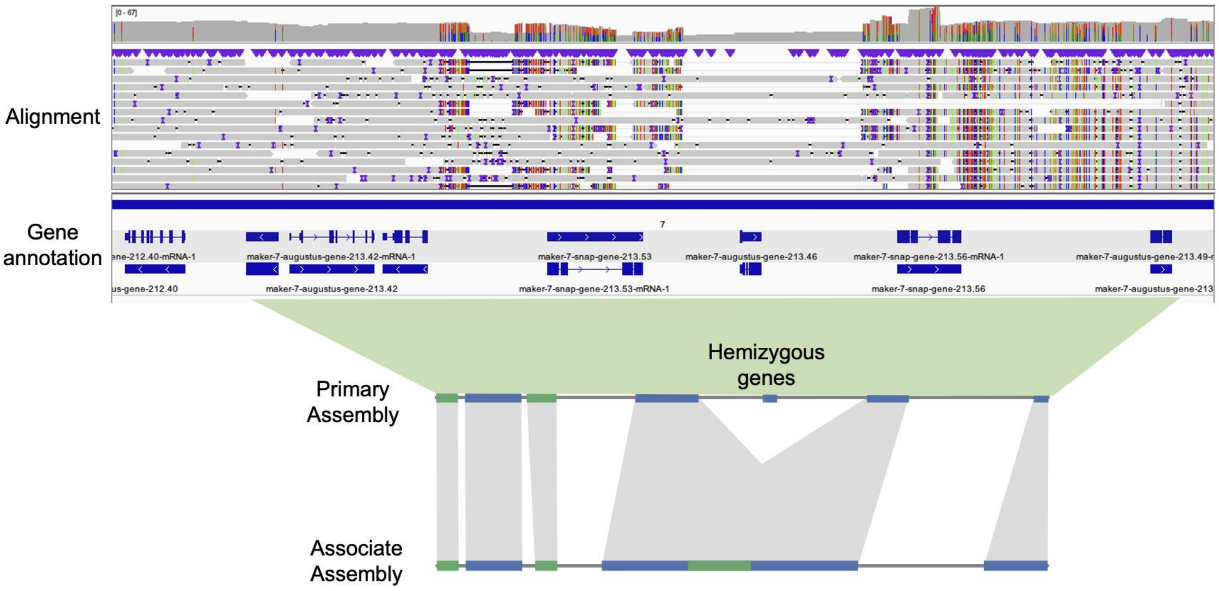

Supplementary Figure S8. A hemizygous gene identified by the mapping approach is also validated by collinear gene analysis between the *V. labrusca* genome primary assembly and associate assembly.

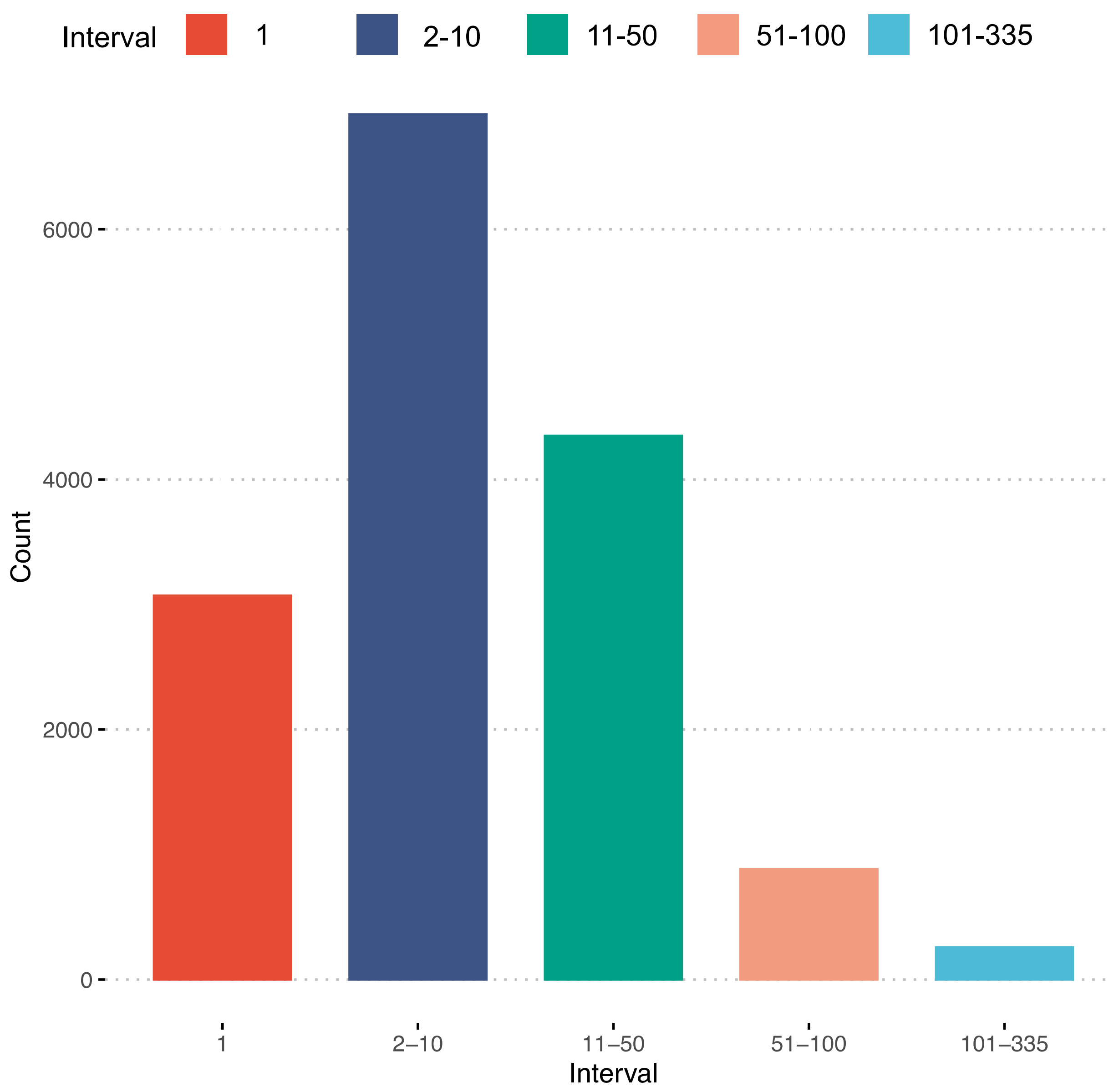

Supplementary Figure S9. Distribution of SNP count within each gene body in the *V. labrusca* genome. In the bar plot, the x-axis indicates different intervals of SNP numbers, from only 1 SNP per gene to 335 SNPs in one gene. The y-axis represents the number of genes that contain these numbers of SNPs.

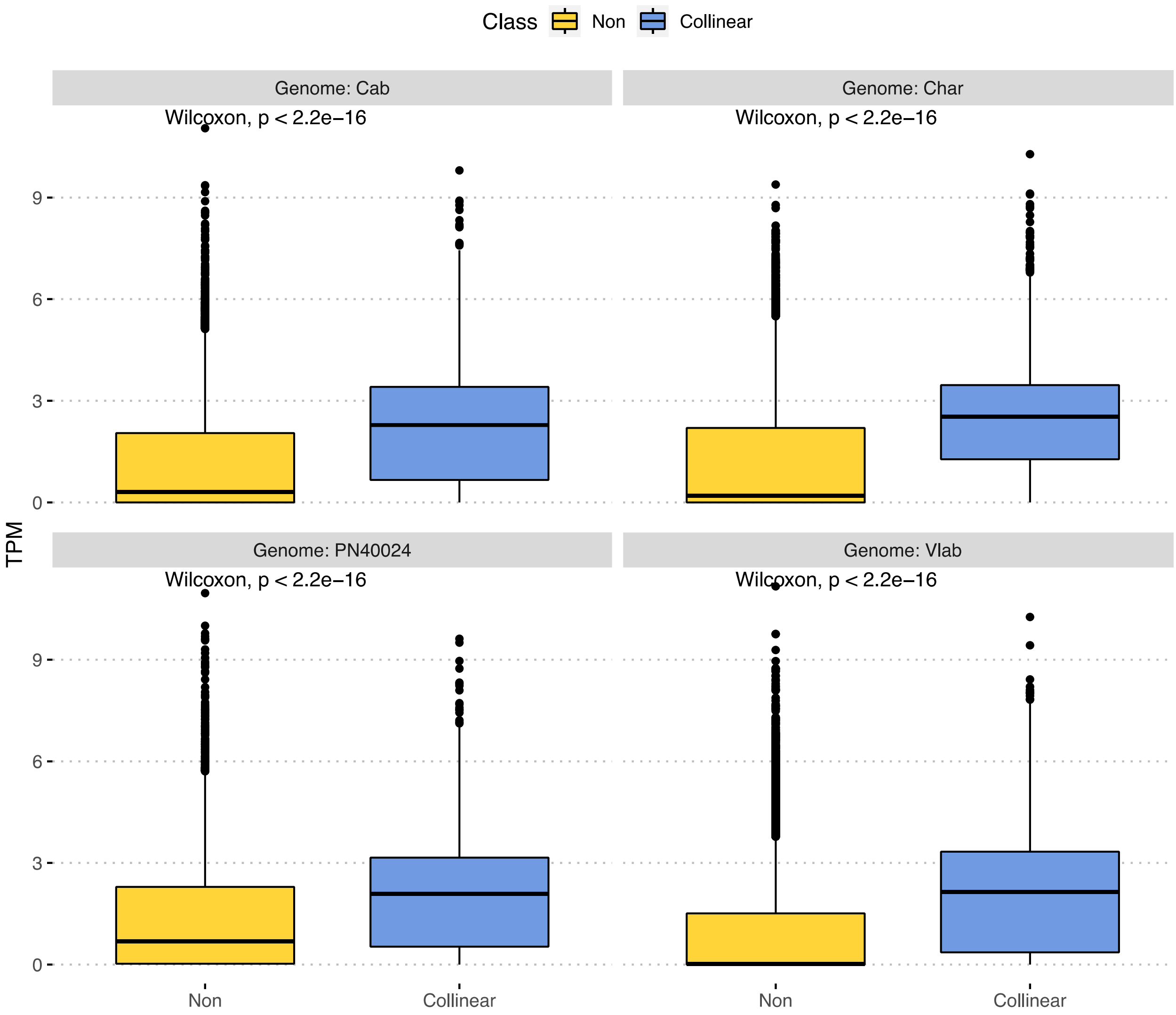

Supplementary Figure S10. Collinear and non-collinear (i.e., Non) gene expression comparisons in berry tissues from four grapevines: PN40042, Cabernet Sauvignon (Cab), Chardonnay (Char), *V. labrusca* Grem 4 (Vlab).

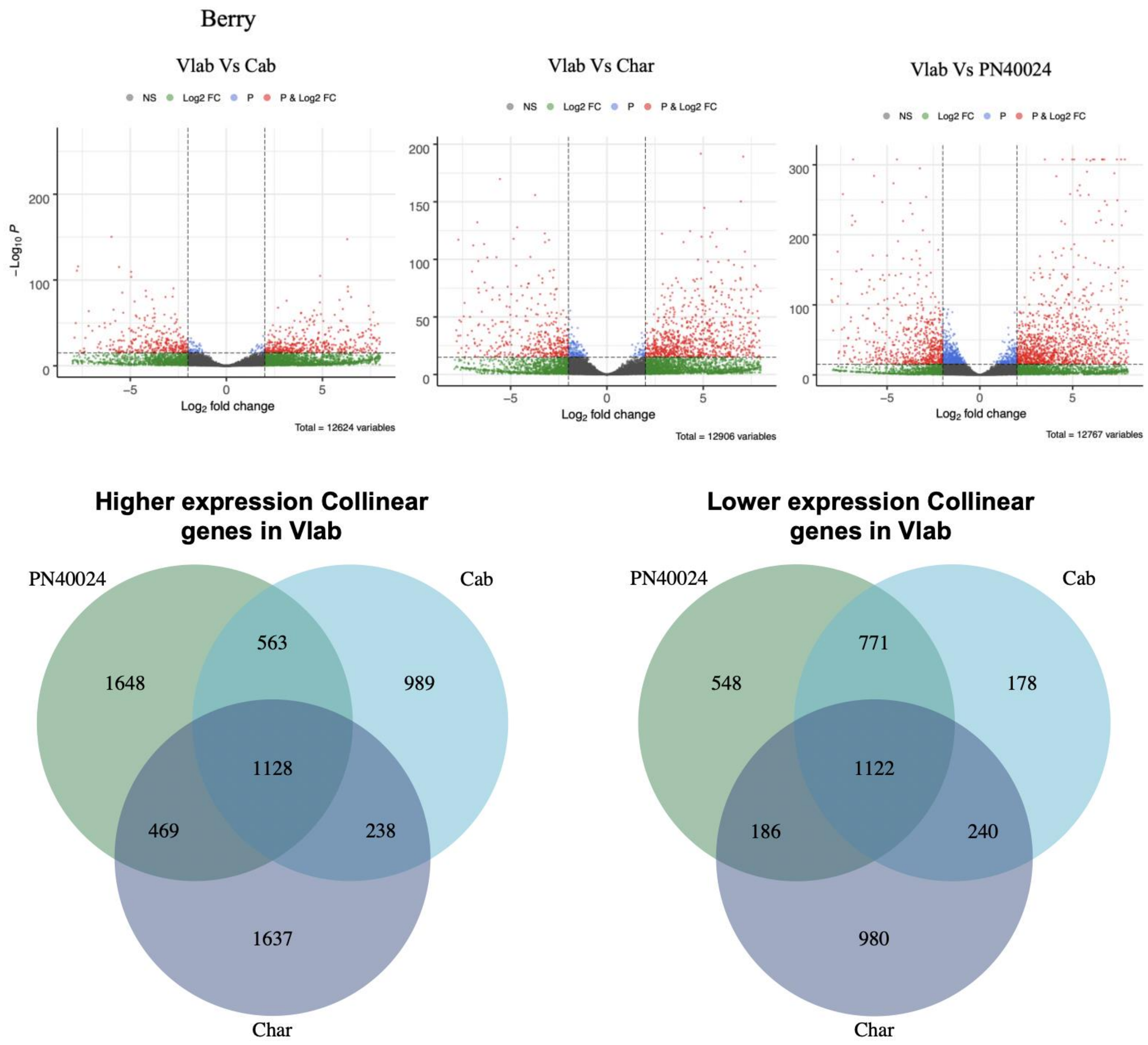

Supplementary Figure S11. Differential expression analysis on collinear genes between *V. labrusca* and three cultivated grapevines. Volcano plots depict pairwise gene expression comparisons in grape berries between *V. labrusca* (veraison) and the other three cultivated grapevine cultivars (pre-veraison to veraison). The Venn diagrams show that expression levels of 1,128 collinear genes were significantly higher in *V. labrusca* than in all cultivated grapes and 1,122 collinear genes showed significantly lower expression in *V. labrusca* compared with other three cultivated grapes.

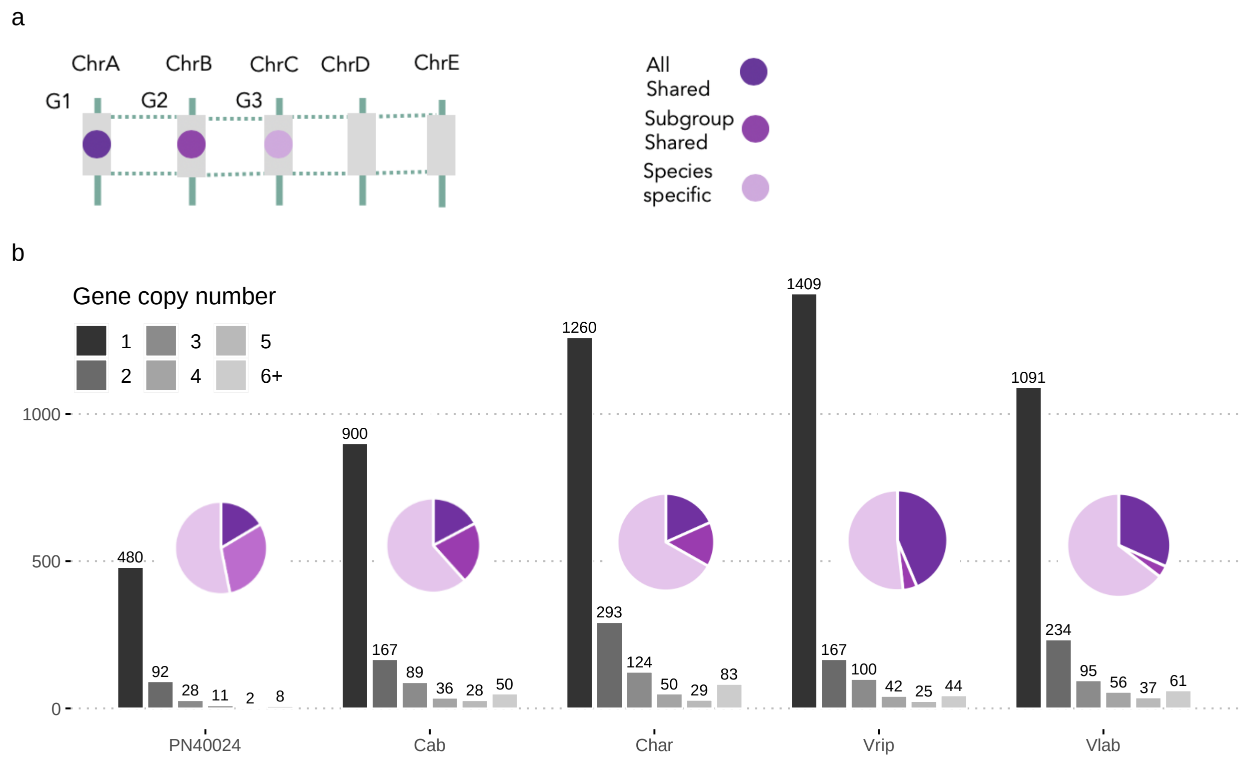

Supplementary Figure S12. a. Segmental duplication can occur multiple times in the same genomic region, which may cause multiple gene duplications (G1, G2 and G3). However, some of these duplicated segments do not have genes, indicating subsequent gene loss after SD (no genes in SD regions on ChrD and ChrE). **b.** Over 70% of segmental duplications from same donor sequence maintained only one gene copy. The bar chart depicts the maintained gene copy numbers in segmentally duplicated regions of the 5 grapevine genomes. Bar colors correspond to gene copy number. The pie chart describes the proportion of All Shared, Subgroup Shared, and Specie-Specific genes in the single copy gene group (dark color bar).

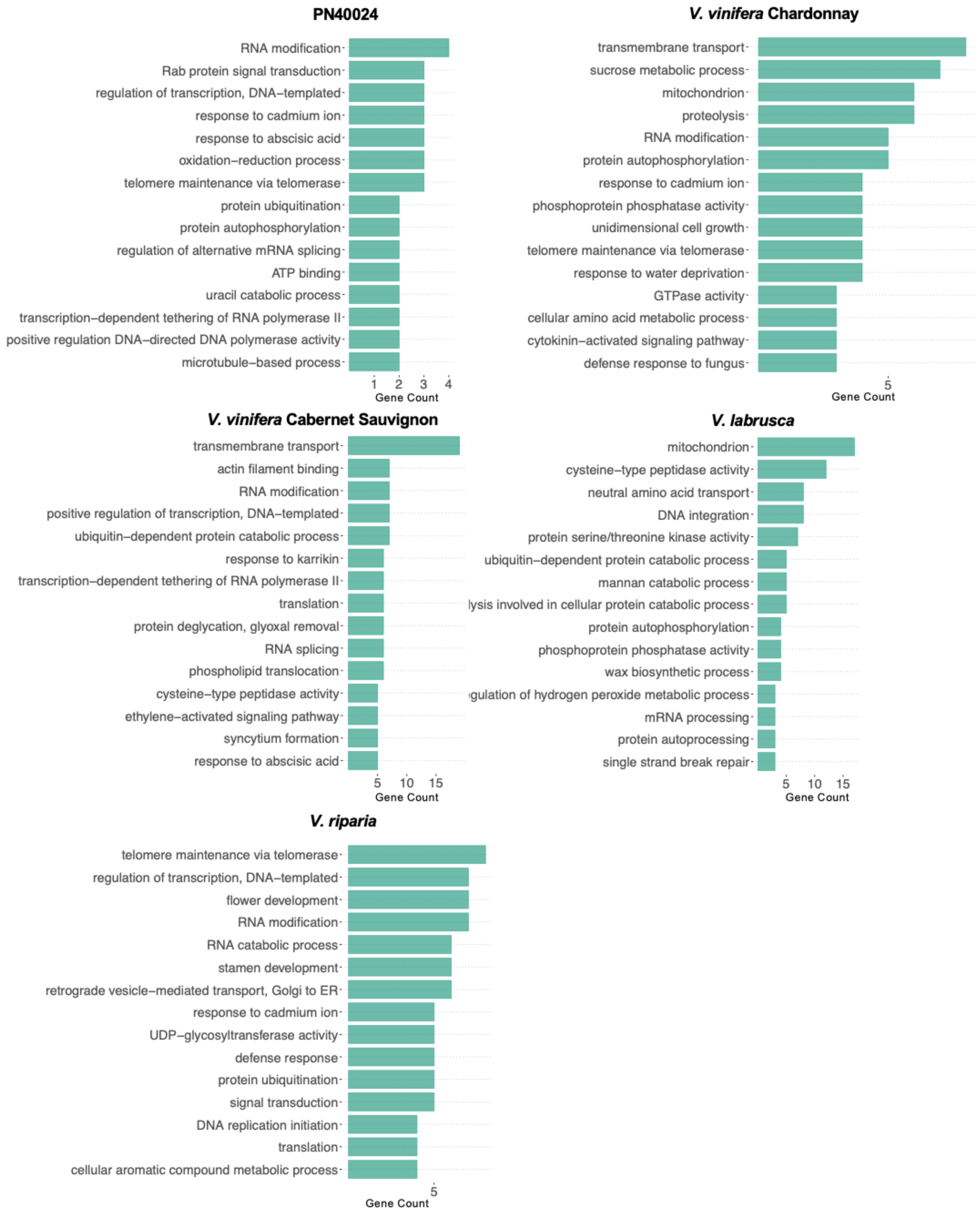

Supplementary Figure S13. Top 15 representative biological functions of the genome-specific segmental duplicated (SD) genes for the five grapevine genomes. The number of SD genes with each function is denoted by the bar and the SD gene count is on the X axis.

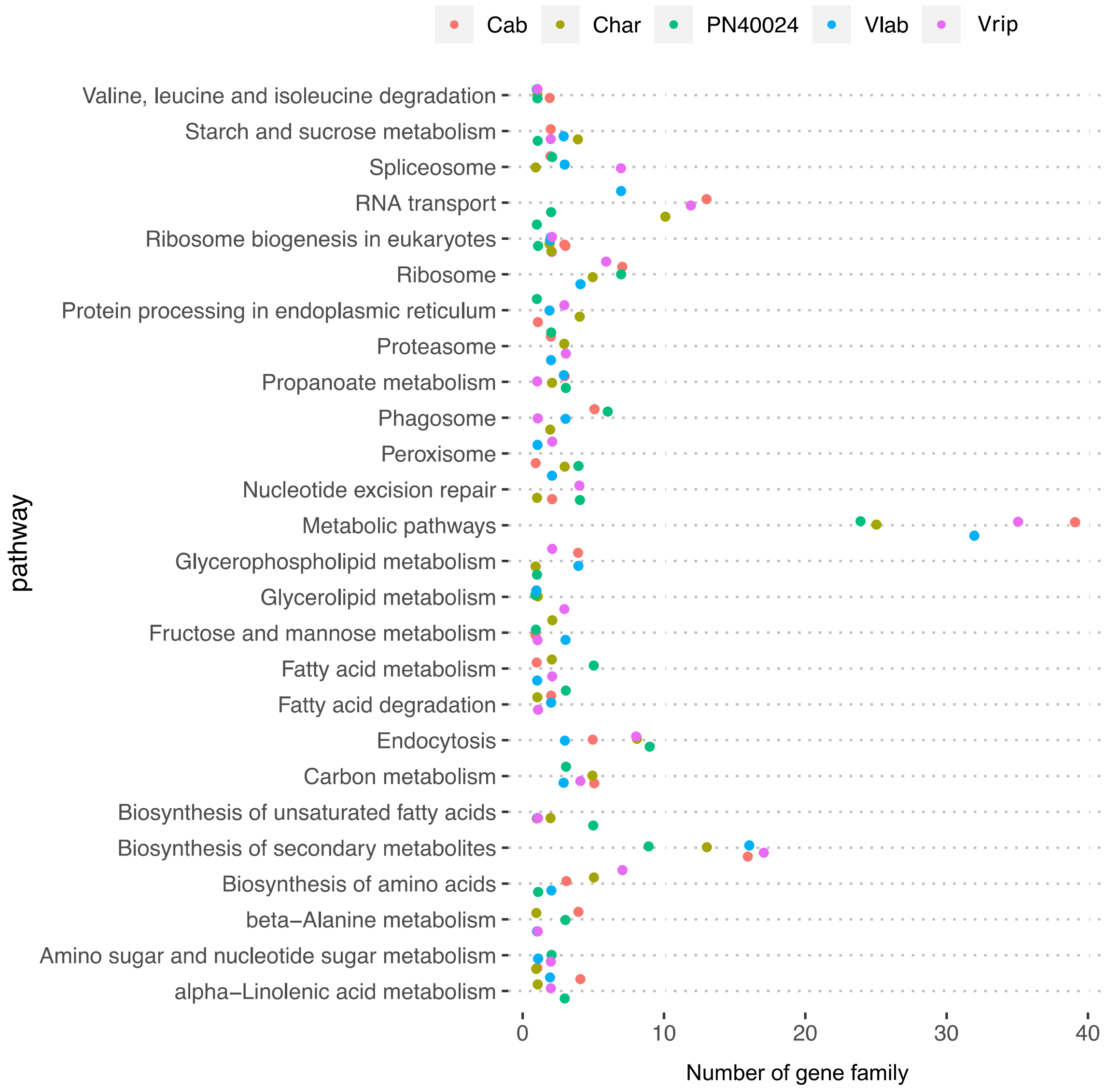

Supplementary Figure S14. Different gene families and duplicated gene count involved in commonly shared KEGG pathways among the five grapevines. The x-axis represents the variable number of gene families affected by SDs in each KEGG pathway among five grapevine genomes and the y-axis represents the KEGG pathways enriched with SD genes in all five grape genomes. PN40042, Cabernet Sauvignon (Cab), Chardonnay (Char), *V. labrusca* Grem 4 (Vlab), and *V. riparia* (Vrip).

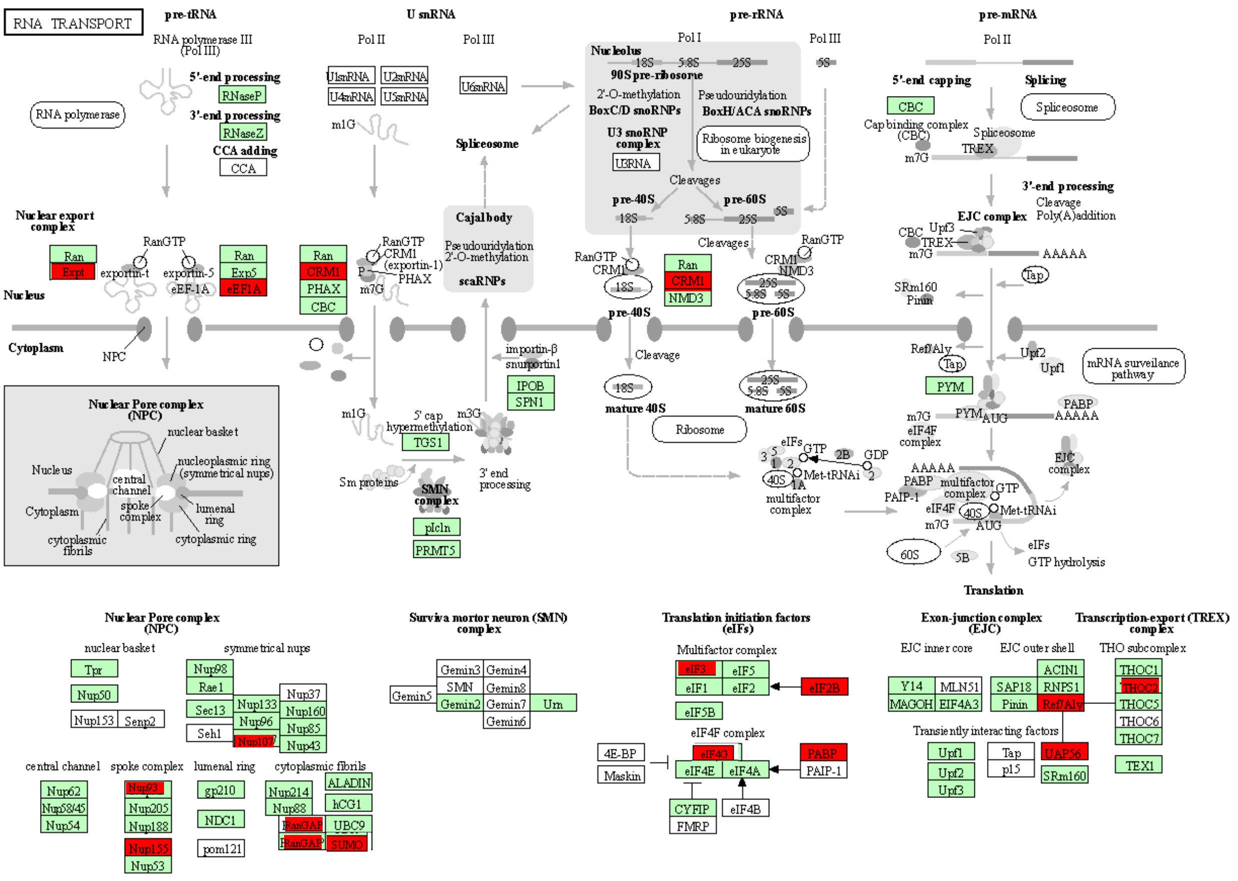

	PN	Cab	Char	Vrip	Vlab
Expt		×			
eEF1A		×	×	×	×
CRM1	×		×	×	×
Nup107		×			
Nup93		×		×	
Nup155	×	×		×	×
RanGAP		×	×	×	

	PN	Cab	Char	Vrip	Vlab
RanBP2					×
SUMO				×	
eIF3		×			×
eIF2B		×			×
EIF4G		×			
PABP		×	×		×
THOC2		×			×
UAP56					×

Supplementary Figure S15. Details about differential amplification and retention of gene families involved in the RNA transport pathway in different grapevine genomes. The red boxes highlight the node genes in the pathway, which were amplified by segmental duplication. The table lists key gene families that were amplified via segmental duplication in the RNA transport pathway in different grapevine genomes. PN40042 (PN), Cabernet Sauvignon (Cab), Chardonnay (Char), *V. riparia* (Vrip), and *V. labrusca* Grem 4 (Vlab).

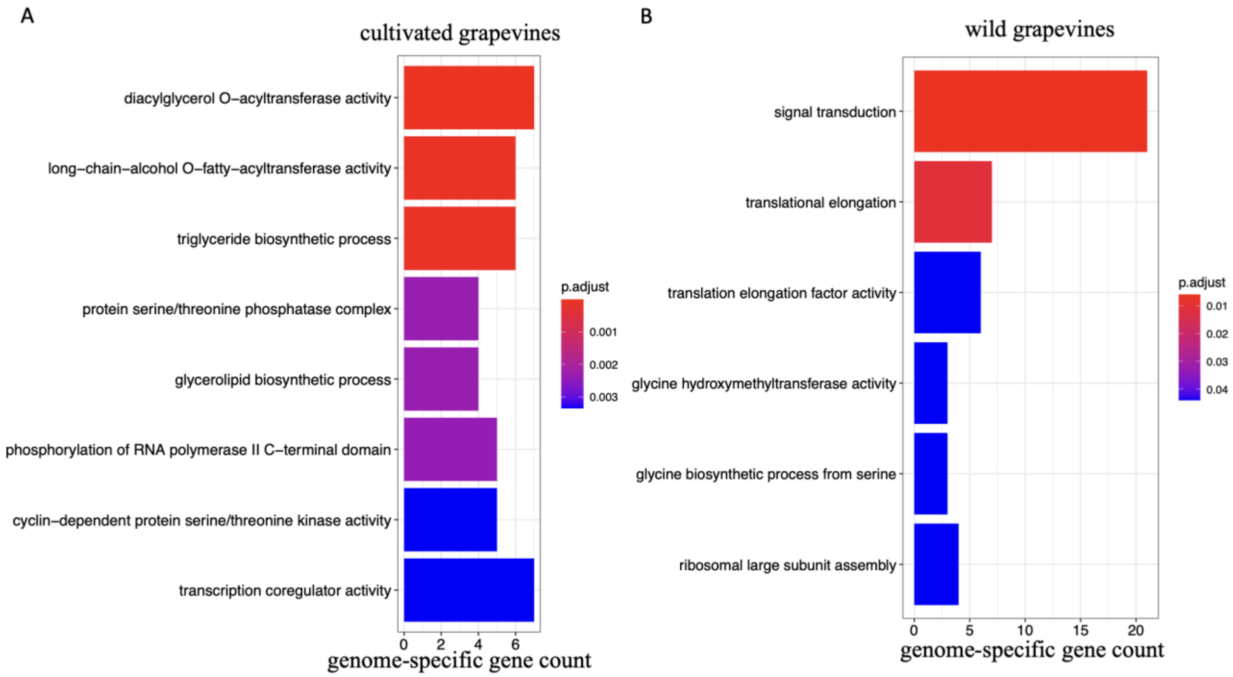

Supplementary Figure S16. Functional annotation of genome-specific genes for cultivated and wild grapevines. A) cultivated grapevines and B) wild grapevines. The gene datasets used for gene ontology enrichment analysis were comprised of both genome-specific gene clusters and singletons.

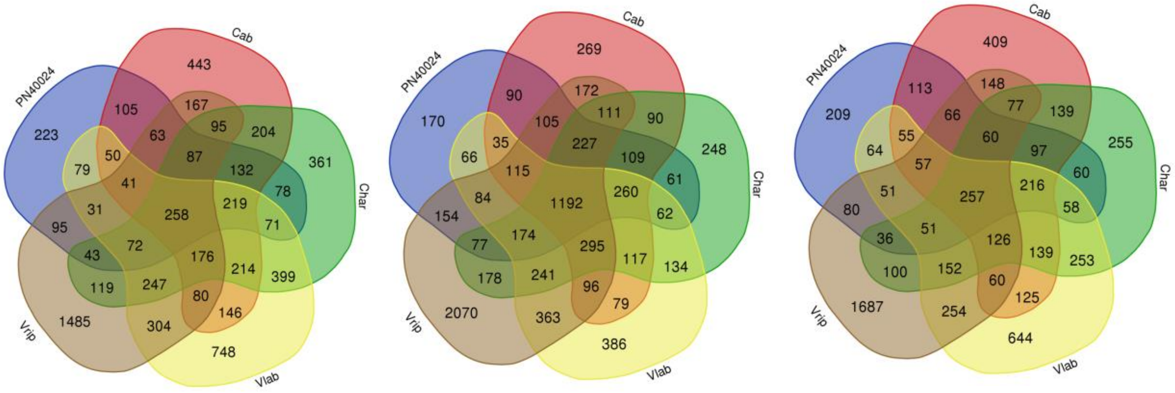

1kb upstream　　Gene Body　　1kb downstream

Supplementary Figure S17. Shared/specific LTR insertions identified in three different genomic regions (1 kb upstream regions of the transcription start site, gene body, and 1 kb downstream regions of the transcription stop site) around collinear genes among the five grapevine genomes.

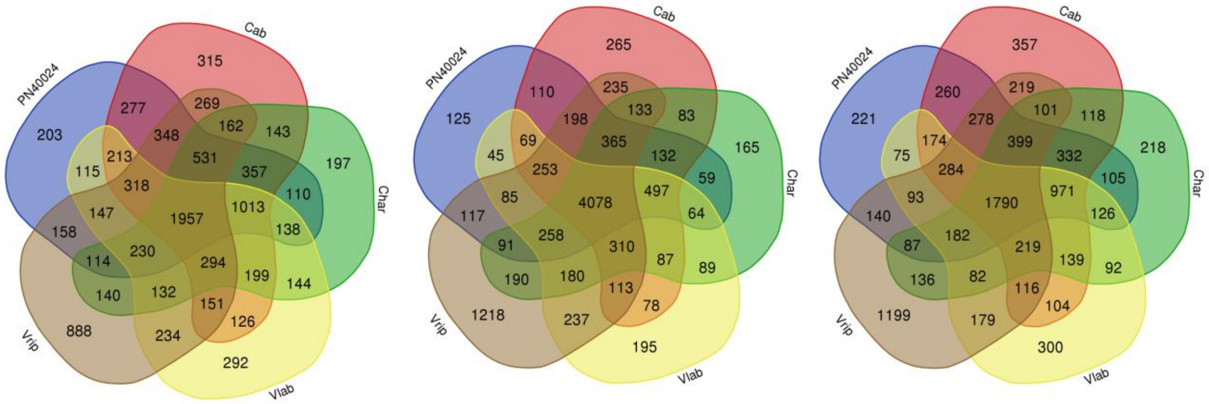

1kb upstream | Gene Body | 1kb downstream

Supplementary Figure S18. Shared/specific MITE insertions identified in three different genomic regions (1 kb upstream regions of the transcription start site, gene body, and 1 kb downstream regions of the transcription stop site) around collinear genes among the five grapevine genomes.

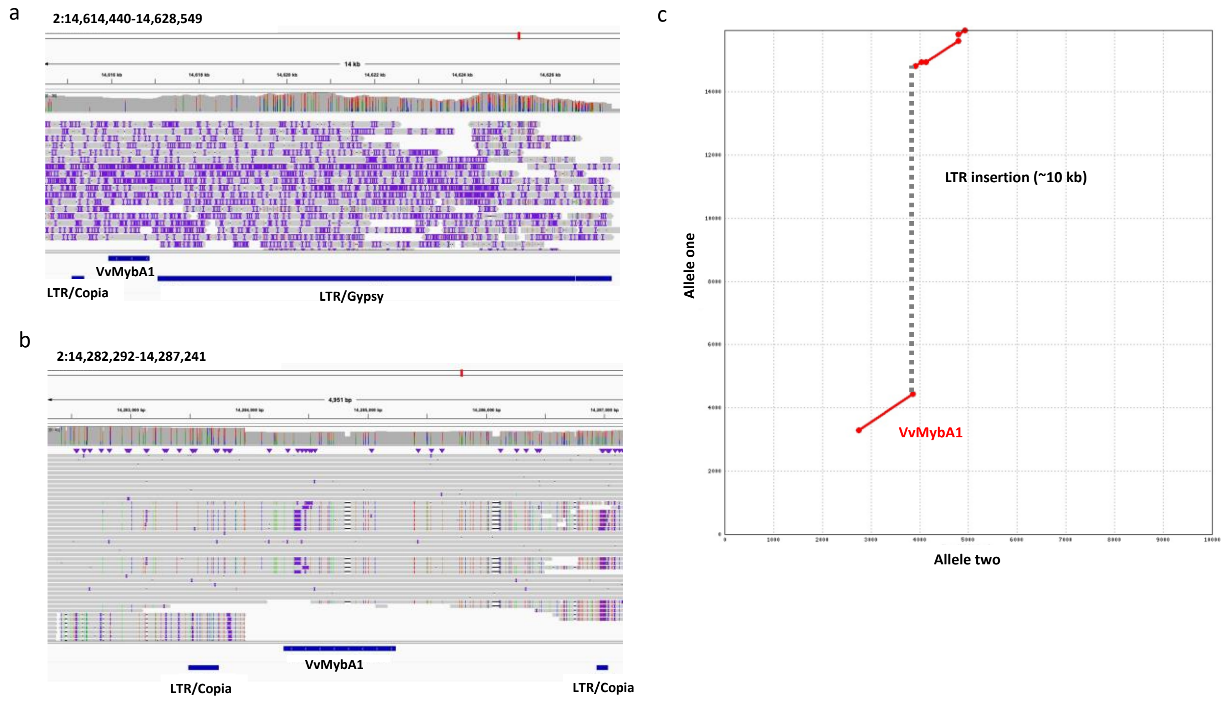

Supplementary Figure S19. TE insertion in different grapevine genomes. **a.** LTR/Gypsy inserted in the upstream region of VvMybA1 in the Chardonnay genome. **b.** LTR insertion of VvMybA1 is absent from the *V. labrusca* genome. **c.** Heterozygous locus of VvMybA1 in Cabernet Sauvignon. The alignment of VvMybA1 sequences extracted from the two haplotype assemblies of Cabernet Sauvignon.
