# Supplementary material for: Vitis labrusca genome assembly reveals diversification between wild and cultivated grapevine genomes
Source: Front Plant Sci. 2023 Aug 31;14:1234130. doi: 10.3389/fpls.2023.1234130 (PMC10501149; doi:10.3389/fpls.2023.1234130)
Supplement: Supplementary file 2 [file DataSheet_2.pdf]

Supplementary Table S1. Summary of PacBio sequencing. Subreads from a total of six PacBio SMRT Cells were generated and summarized with some key statistics to show the sequencing quality. The top 8 rows are the statistics for total reads and the bottom 8 rows are for longest subreads only: the total number of subreads (numReadsSubread), the total bases of subreads (totalBasesSubread), mean length of subreads (meanReadLenSubread), median read length of subreads (medianReadLenSubread), N50 of subreads (n50Subread), I50 of subread (I50Subread).

SMRT cell	numReads Subread	totalBases Subread (bp)	meanRead LenSubread (bp)	medianRead LenSubread (bp)	n50Subread (bp)	l50Subread (bp)
Cell1	1615004	9953181724	6163	2181	19838	148754
Cell2	1548205	10103972299	6526	2495	19679	153552
Cell3	1313760	8208331822	6248	2283	19382	126232
Cell4	1617122	10706601913	6621	2624	19424	163973
Cell5	1547469	11550882570	7464	2866	21902	165166
Cell6	1208617	7946815346	6575	2245	21343	114310
Total	8850177	58469785674	6599	2449	20261	871987
**SMRT cell**	**numReads LongestSub**	**totalBases LongestSub (bp)**	**meanRead LenLongestS ub (bp)**	**medianRead LenLongestS ub (bp)**	**n50LongestS ub (bp)**	**l50 LongestSub (bp)**
Cell1	344242	5505703302	15994	11123	28968	66740
Cell2	387205	5953583300	15376	10739	27583	74913
Cell3	318932	4848175622	15201	10576	27356	61516
Cell4	392737	6077094864	15474	10769	27742	76146
Cell5	414393	6866556458	16570	11976	29263	83209
Cell6	284051	4643323342	16347	11545	29517	55730
Total	2141560	33894436888	15827	11121	28404	418254

Supplementary Table S2. Summary of genome assembly.

Assembly main steps	Major statistics	Primary contigs (Vlab_PG) (bp)	Associate contigs (Vlab_AG) (bp)
(1) Falcon-unzip assembly	Contig No.	669	1846
	Largest contig	10,188,842	1,617,434
	Mean length of contigs	848,777	183,266
	Median length of contigs	321,275	112,592
	Contig N50	2,079,183	322,470
	Contig N95	204,632	52,788
	Total assembly	567,832,413	338,309,457
(2) Purge Haplotigs correction	Contig No.	295	2,220
(3) Reference based validation on Purge Haplotigs results	Contig No.	438 (+143)[a]	2,077
	Contig N50	2,526,427	331,772
	Contig N90	583,620	93,366
	Total assembly	502,100,454	404,041,416

a: 143 contigs were assigned back to the primary assembly.

Supplementary Table S3. Genome summary of the *V. labrusca* pseudomolecules.

#chr	Primary assembly (Vlab_PG)			Associate assembly (Vlab_AG)			
	Total Size (bp)	Contig Number	GC content	Total Size (bp)	Contig Number	GC content	Length difference (bp)
**1_labrusca**	23,119,760	11	0.34	21,422,276	95	0.34	1,697,484
**2_labrusca**	20,186,406	13	0.34	16,623,823	86	0.34	3,562,583
**3_labrusca**	20,721,644	17	0.34	15,051,897	68	0.34	5,669,747
**4_labrusca**	24,672,639	13	0.34	20,673,311	97	0.34	3,999,328
**5_labrusca**	23,490,163	13	0.35	19,099,203	109	0.35	4,390,960
**6_labrusca**	21,972,982	12	0.34	20,191,201	110	0.34	1,781,781
**7_labrusca**	24,840,418	9	0.35	23,831,987	105	0.34	1,008,431
**8_labrusca**	22,435,566	11	0.34	21,092,394	92	0.35	1,343,172
**9_labrusca**	23,523,929	24	0.34	23,428,916	112	0.34	95,013
**10_labrusca**	21,936,408	17	0.34	19,697,660	92	0.34	2,238,748
**11_labrusca**	21,005,569	10	0.34	17,923,055	84	0.34	3,082,514
**12_labrusca**	24,794,315	19	0.34	21,200,977	112	0.34	3,593,338
**13_labrusca**	32,554,736	24	0.34	23,492,953	106	0.34	9,061,783
**14_labrusca**	30,182,383	23	0.34	24,599,146	144	0.34	5,583,237
**15_labrusca**	22,349,701	12	0.34	15,417,736	81	0.34	6,931,965
**16_labrusca**	24,060,753	31	0.34	20,078,384	103	0.34	3,982,369
**17_labrusca**	14,314,453	11	0.35	11,731,294	58	0.35	2,583,159
**18_labrusca**	33,779,012	31	0.35	26,653,749	132	0.35	7,125,263
**19_labrusca**	26,432,707	21	0.34	19,447,158	110	0.34	6,985,549
**Pseudomolecule[1]**	456,373,544	322	0.34	381,657,120	1896	0.34	74,716,424
**ref_unassembly[2]**	38,904,312	89	0.35	19,253,511	158	0.35	19,650,801
**labrusca_specific contigs[3]**	6,863,598	27	0.38	3,334,785	23	0.39	3,528,813
**Total assembly[4]**	502,141,454	438	0.34	404,245,416	2077	0.34	97,896,038

1.Contigs that could be scaffolded into chromosomes; 2. Contigs that could align to PN40024 contigs but could not be scaffolded into chromosomes; 3. Contigs that could not be aligned to the PN40024 reference. 4. The total length of all scaffolds (which contains the length of all contigs and 100 Ns for each gap).

Supplementary Table S4. TE annotation summary in the *V. labrusca* genome.

Repeat Class	Number of elements	Total length (bp)	Percentage of genome (%)
Total TE	567,896	250,054,547	49.8
RNA retroelements	217,909	135,246,080	26.93
LTR/Copia	40,629	18,133,666	3.61
LTR/Gypsy	65,875	61,608,669	12.27
LTR/Unknown	111,405	55,503,745	11.05
DNA transposon	212,539	38,645,527	7.7
DTA(hAT)	13,901	2,506,853	0.4992
DTC(CACTA)	1,330	255,507	0.0509
DTH(Pif/Harbinger)	44,965	9,197,642	1.8317
DTM(Mutator)	43,897	11,044,876	2.1996
DTT(Tc1/Mariner)	108,446	15,640,649	3.1148
Helitron	27	4,580	0.0009
unknown	137,448	76,162,940	15.17

Supplementary Table S5. rRNA loci identification in *V. labrusca* and other grapevine genomes.

	**PN40024**	**Cabernet Sauvignon**	**Chardonnay**	***V. riparia***	***V. labrusca***
5S CN	35	59	49	17	49
Chromosome locations of 5S gene cluster	chr17	chr17	chr6, chr17	chr5, chr17	chr17
5.8S CN[a]	2	57	4	1	8
18S CN	9	77	14	9	15
25S CN	15	79	25	8	18
Chromosome locations of 45S gene cluster	Un, chr15,	Un, chr15	chr15	chr4, chr6, chr10, chr15,	chr15 and chr19

a: 45S copy and chromosome location are identified with 5.8S, 18S and 25S, independently.
Un= unassembled contigs.

Supplementary Table S6. The overall estimation of intragenomic sequence variation in the *V. labrusca* genome.

	Total bases of primary genome	Total bases of associate genome	Aligned bases	Unaligned bases	Proportion of unaligned bases	Proportion of dissimilar bases within aligned regions	Overall sequence variation
**Chr1**	23119760	21422276	20909447	512829	2.39%	1.13%	3.52%
**Chr2**	20186406	16623823	16236725	387098	2.33%	2.70%	5.03%
**Chr3**	20721644	15051897	14553868	498029	3.31%	1.81%	5.12%
**Chr4**	24672639	20673311	20141714	531597	2.57%	1.25%	3.82%
**Chr5**	23490163	19099203	18492811	606392	3.17%	1.40%	4.57%
**Chr6**	21972982	20191201	19725344	465857	2.31%	1.33%	3.64%
**Chr7**	24840418	23831987	23363458	468529	1.97%	1.21%	3.18%
**Chr8**	22435566	21092394	20320083	772311	3.66%	1.36%	5.02%
**Chr9**	23523929	23428916	21924427	1504489	6.42%	2.36%	8.78%
**Chr10**	21936408	19697660	18778674	918986	4.67%	1.99%	6.66%
**Chr11**	21005569	17923055	17478285	444770	2.48%	1.17%	3.65%
**Chr12**	24794315	21200977	20376464	824513	3.89%	2.22%	6.11%
**Chr13**	32554736	23492953	22576453	916500	3.90%	1.91%	5.81%
**Chr14**	30182383	24599146	23993768	605378	2.46%	1.68%	4.14%
**Chr15**	22349701	15417736	14549037	868699	5.63%	1.95%	7.58%
**Chr16**	24060753	20078384	19227010	851374	4.24%	2.20%	6.44%
**Chr17**	14314453	11731294	11591244	140050	1.19%	1.00%	2.19%
**Chr18**	33779012	26653749	25887346	766403	2.88%	1.36%	4.24%
**Chr19**	26432707	19447158	18743874	703284	3.62%	1.71%	5.33%
**Overall intragenomic variation**							**4.99%**

Supplementary Table S7. The overall estimation of intragenomic sequence variation in the Chardonnay genome.

	Total bases of primary genome	Total bases of associate genome	Aligned bases	Unaligned bases	Proportion of unaligned bases	Proportion of dissimilar bases within aligned regions	Overall sequence variation
**Chr1**	24116249	19961854	18946634	1015220	5.09%	2.50%	7.59%
**Chr2**	19011114	10576800	9001584	1575216	14.89%	3.31%	18.20%
**Chr3**	20547032	13116730	12382487	734243	5.60%	3.15%	8.75%
**Chr4**	25864336	21182098	20424558	757540	3.58%	2.44%	6.02%
**Chr5**	28416557	20189109	19117812	1071297	5.31%	2.85%	8.16%
**Chr6**	22566232	17863405	17181494	681911	3.82%	2.46%	6.28%
**Chr7**	18733084	16786706	14242397	2544309	15.16%	2.94%	18.10%
**Chr8**	24260300	19918391	18934101	984290	4.94%	2.93%	7.87%
**Chr9**	22310039	16976042	15764026	1212016	7.14%	3.16%	10.30%
**Chr10**	19111643	17210919	15759264	1451655	8.43%	2.81%	11.24%
**Chr11**	20195324	15426607	14760481	666126	4.32%	2.41%	6.73%
**Chr12**	23073756	17983869	15947902	2035967	11.32%	3.14%	14.46%
**Chr13**	27103049	17581193	16219625	1361568	7.74%	3.02%	10.76%
**Chr14**	33843232	24825739	23326850	1498889	6.04%	3.04%	9.08%
**Chr15**	21751509	15461934	14471991	989943	6.40%	3.21%	9.61%
**Chr16**	24103225	17745947	16725360	1020587	5.75%	2.92%	8.67%
**Chr17**	18320282	13276092	12580391	695701	5.24%	3.22%	8.46%
**Chr18**	28524900	25806860	23825887	1980973	7.68%	2.92%	10.60%
**Chr19**	25259163	21984664	20316212	1668452	7.59%	2.98%	10.57%
**Overall intragenomic variation**							**10.08%**

Supplementary Table S8. The overall estimation of intragenomic sequence variation in the Cabernet Sauvignon genome.

	Total bases of primary genome	Total bases of associate genome	Aligned bases	Unaligned bases	Proportion of unaligned bases	Proportion of dissimilar bases within aligned regions	Overall sequence variation
Chr1	21969846	19414891	16530325	2884566	14.86%	2.32%	17.18%
Chr2	20217481	20558208	18134551	2423657	11.79%	2.43%	14.22%
Chr3	18253582	19665921	16090480	3575441	18.18%	3.23%	21.41%
Chr4	22679887	23046113	19005321	4040792	17.53%	2.14%	19.67%
Chr5	25461128	25785018	22555717	3229301	12.52%	2.96%	15.48%
Chr6	23567781	20762368	18609535	2152833	10.37%	2.76%	13.13%
Chr7	29508925	26684737	23800509	2884228	10.81%	2.84%	13.65%
Chr8	24360825	21896409	19728934	2167475	9.90%	2.68%	12.58%
Chr9	24550656	22112190	19623421	2488769	11.26%	3.26%	14.52%
Chr10	24240781	21792205	18388621	3403584	15.62%	2.86%	18.48%
Chr11	19351447	15936280	13939612	1996668	12.53%	3.11%	15.64%
Chr12	26052447	24651902	21621054	3030848	12.29%	2.60%	14.89%
Chr13	28968403	25535923	20837414	4698509	18.40%	2.36%	20.76%
Chr14	27309586	27254004	22641204	4612800	16.93%	2.80%	19.73%
Chr15	21170564	20202138	17689175	2512963	12.44%	3.24%	15.68%
Chr16	23182680	22263809	18777630	3486179	15.66%	3.36%	19.02%
Chr17	17785969	18180427	15202806	2977621	16.38%	2.81%	19.19%
Chr18	36594081	34107272	29801070	4306202	12.63%	2.67%	15.30%
Chr19	24755257	24161115	19534965	4626150	19.15%	2.87%	22.02%
**Overall intragenomic variation**							**16.98%**

Supplementary Table S9. The overall estimation of intragenomic sequence variation in the *V. vinifera* ssp. *sylvestris* genome.

	Total bases of primary genome	Total bases of associate genome	Aligned bases	Unaligned bases	Proportion of unaligned bases	Proportion of dissimilar bases within aligned regions	Overall sequence variation
Chr1	34956132	18288296	17901197	387099	2.12%	1.79%	3.91%
Chr2	13662549	8050940	7275480	775460	9.63%	1.81%	11.44%
Chr3	22302269	1248062<br>9	10770188	1710441	13.70%	2.03%	15.73%
Chr4	32713915	1862120<br>0	17877971	743229	3.99%	1.57%	5.56%
Chr5	41861358	16935465	16405207	530258	3.13%	1.70%	4.83%
Chr6	27684767	14726151	13060193	1665958	11.31%	1.64%	12.95%
Chr7	25596386	14262450	11611101	2651349	18.59%	1.75%	20.34%
Chr8	36118687	16606040	16344268	261772	1.58%	1.58%	3.16%
Chr9	28174022	12171476	10919400	1252076	10.29%	1.78%	12.07%
Chr10	27208680	11091046	10706919	384127	3.46%	1.91%	5.37%
Chr11	32719217	13020357	12636616	383741	2.95%	1.90%	4.85%
Chr12	31187245	15398923	14646922	752001	4.88%	1.68%	6.56%
Chr13	37564735	17119332	1658705<br>2	532280	3.11%	1.70%	4.81%
Chr14	43990609	19670329	19234970	435359	2.21%	1.71%	3.92%
Chr15	24909400	13258349	11860117	1398232	10.55%	2.11%	12.66%
Chr16	36236122	15874640	15402683	471957	2.97%	1.60%	4.57%
Chr17	28092373	9666891	9489260	177631	1.84%	1.60%	3.44%
Chr18	36818813	20152276	19049416	1102860	5.47%	1.65%	7.12%
Chr19	38416532	15033403	14486830	546573	3.64%	1.84%	5.48%
**Overall intragenomic variation**							**7.83%**

Supplementary Table S10. Hemizygous genes calculated within each deletion region.

	**The number of deletions containing genes**	**Hemizygous gene number***
**Total**	**663**	**1,756**
**<10Kb**	304	377
**10-20Kb**	118	186
**20-100Kb**	188	517
**>100Kb**	53	676

*Note: hemizygous genes shared by more than one SV region have been removed from larger SV groups. Deletions without affecting genes are not included.

Supplementary Table S11. Functional annotation of fourteen single copy hemizygous genes in the *V. labrusca* genome.

SingleCopyHemigene	Uniprot Gene ID	GO Term	GO name	GO Category
**maker-1-augustus-gene-42.39-mRNA-1**	A5AYU9	GO:0055114	oxidation-reduction process	biological_process
**maker-16-snap-gene-122.29-mRNA-1**	W1NRR0	GO:0006412	translation	biological_process
**snap-18-processed-gene-260.15-mRNA-1**	D7T9L3	GO:0045893	positive regulation of transcription, DNA-templated	biological_process
**maker-5-snap-gene-194.59-mRNA-1**	F6I3S6	GO:0019752	carboxylic acid metabolic process	biological_process
**maker-19-augustus-gene-115.18-mRNA-1**	D7UAQ6	GO:0006281	DNA repair	biological_process
**augustus-1-processed-gene-69.6-mRNA-1**	A5BAM7	GO:0055114	oxidation-reduction process	biological_process
**maker-5-snap-gene-195.66-mRNA-1**	F6I3S8	GO:0046621	negative regulation of organ growth	biological_process
**maker-16-augustus-gene-14.27-mRNA-1**	F6HVB1	GO:0007623	circadian rhythm	biological_process
**maker-5-augustus-gene-194.42-mRNA-1**	F6I3S3	GO:0000245	spliceosomal complex assembly	biological_process
**snap-5-processed-gene-98.14-mRNA-1**	A0A2J6KJV1	GO:0055114	oxidation-reduction process	biological_process
**maker-19-snap-gene-115.19-mRNA-1**	D7UAQ7	GO:0046686	response to cadmium ion	biological_process
**maker-17-snap-gene-85.23-mRNA-1**	D7SHE7	GO:0000712	resolution of meiotic recombination intermediates	biological_process
**snap-14-processed-gene-249.20-mRNA-1**	F6HXB6	GO:0005975	carbohydrate metabolic process	biological_process
**maker-16-snap-gene-16.26-mRNA-1**	F6HVA7	GO:0000077	DNA damage checkpoint	biological_process

Supplementary Table S12. Heterozygous genes caused by SVs (31- 859,735 bp) identified between homologous chromosomes in *V. labrusca*.

SV_type	Genomic_feature	Count
**Indel**	Total	12410
**Insertion**	Total	4342
	Intergenic region	3211
	Intron	1045
	Exon	86
**Deletion**	Total	8068
	Intergenic region	5359
	Intron	1960
	Exon	749
**Heterozygous genes (gene level)[a]**		2919
**Heterozygous genes (protein level)[b]**		690

a: Heterozygous genes at the gene level are defined as having an indel occurring within an intron, exon, or both.
b: Heterozygous genes at the protein level are defined as having an indel that changes the amino acid sequence (occurring within an exon).
Multiple indels are existent in one gene.

Supplementary Table S13. Heterozygous genes caused by Small Indels (2- 30bp) identified between homologous chromosomes in *V. labrusca*.

SV_type	Genomic_feature	Count
**Indel**	Total	26230
**Insertion**	Total	11322
	Intergenic region	8765
	Intron	2251
	Exon	306
**Deletion**	Total	14908
	Intergenic region	11795
	Intron	2642
	Exon	471
**Heterozygous genes (gene level)**[a]		4544
**Heterozygous genes (protein level)**[b]		756

a: Heterozygous genes at the gene level are defined as having a small indel occurring within an intron, exon, or both.
b: Heterozygous genes at the protein level are defined as having a small indel that changes the amino acid sequence (occurring within an exon).
Multiple indels are existent in one gene.

Supplementary Table S14. Heterozygous genes caused by SNPs identified between homologous chromosomes in *V. labrusca*.

	SNP count
**Total SNPs**	2,000,655
**HQ SNPs (after VCFtools filtering)**	605,809
**Intergenic regions**	362,368
**Genic regions**	243,441
**Intron**	191,628
**Exon**	51,813
**SNPs with missense Effect (SnpEff annotation)**	27,868
**Heterozygous Gene Count (gene level)**	16,133
**Heterozygous Gene count (protein level)**	11,359
**Heterozygous Gene Count (protein level with missense annotation)**	8,693

Supplementary Table S15. Heterozygous genes caused by SVs (31- 859,735 bp) identified between homologous chromosomes in Chardonnay.

SV_type	Genomic_feature	Count
**Indel**	Total	32781
**Insertion**	Total	16488
	Intergenic region	12216
	Intron	3977
	Exon	295
**Deletion**	Total	16293
	Intergenic region	11260
	Intron	4262
	Exon	771
**Heterozygous genes (sequence level)**		5650
**Heterozygous genes (protein level)**		854

Supplementary Table S16. Heterozygous genes caused by Small Indels (2-30 bp) identified between homologous chromosomes in Chardonnay.

SV_type	Genomic_feature	Count
**Indel**	Total	49711
**Insertion**	Total	33288
	Intergenic region	24412
	Intron	8247
	Exon	629
**Deletion**	Total	16162
	Intergenic region	11757
	Intron	4186
	Exon	219
**Heterozygous genes (sequence level)**		6315
**Heterozygous genes (protein level)**		736

Supplementary Table S17. Heterozygous genes caused by SNPs identified between homologous chromosomes in Chardonnay.

	**SNP count**
**Total SNPs**	2178574
**HQ SNPs (after VCFtools filtering)**	1255681
**Intergenic regions**	806049
**Genic regions**	449632
**Intron**	366132
**Exon**	83500
**SNPs with missense Effect (SnpEff annotation)**	12521
**Heterozygous Gene Count (SNPs within gene body)**	18401
**Heterozygous Gene count (SNPs within exon)**	15434
**Heterozygous Gene Count (snpEFF annotation with big Effect)**	12521

Supplementary Table S18. Pairwise collinear gene identification among the five grapevine genomes with the PN40024 genome serving as the reference for the collinear genes analyses.

	PN40024	Cabernet Sauvignon	Chardonnay	*V. riparia*	*V. labrusca*
PN40024		20984/5604	21433/5155	20438/6150	21515/5073
Cabernet Sauvignon	27420/9269		28295/8394	27317/9372	28105/8584
Chardonnay	23371/11087	23243/11215		23058/11400	25503/8955
V. riparia	21743/13755	22297/13201	22913/12585		23420/12078
V. labrusca	22367/10745	20518/12594	23227/9885	23579/9533	

Note: (collinear gene count)/(non-collinear gene count).

Supplementary Table S19. Identification of segmental duplications in the *V. labrusca* genome.

chr	1-5k	Mean identity between SD regions	5-10k	Mean identity between SD regions	10-20k	Mean identity between SD regions	>20k	Mean identity between SD regions	Duplicated region length (bp)	Total number of SD
**1**	1340	92.77	124	93.74	16	95.19	3	98.70	3,659,552	1,483
**2**	1463	92.56	112	94.56	18	95.14	5	98.58	3,876,887	1,598
**3**	1842	92.66	164	93.87	25	94.85	6	98.90	5,103,108	2,037
**4**	1738	92.70	167	93.78	24	94.34	4	99.38	4,825,458	1,933
**5**	1666	92.59	132	93.77	8	94.45	2	99.00	4,215,521	1,808
**6**	1344	92.56	114	94.05	9	95.36	5	98.98	3,557,670	1,472
**7**	1567	92.55	125	94.16	14	94.82	1	97.64	3,995,207	1,707
**8**	1263	92.67	106	94.29	7	94.99	3	98.77	3,252,369	1,379
**9**	1780	92.86	158	93.99	24	94.20	2	97.98	4,868,794	1,964
**10**	1694	92.62	144	93.70	17	94.43	3	99.32	4,444,488	1,858
**11**	1570	92.77	123	94.25	16	96.11	9	98.46	4,208,540	1,718
**12**	1931	92.62	178	94.12	25	95.28	10	97.58	5,559,265	2,144
**13**	2387	92.79	305	94.33	46	95.75	17	98.13	7,754,468	2,755
**14**	2227	92.56	193	93.84	19	94.82	7	99.03	5,958,906	2,446
**15**	1893	92.75	173	93.95	20	94.39	7	99.16	5,244,510	2,093
**16**	2010	92.70	162	94.38	24	95.26	4	99.44	5,291,520	2,200
**17**	968	92.66	95	94.10	28	94.59	7	98.33	3,070,788	1,098
**18**	2824	92.67	220	94.18	29	95.15	4	98.57	7,343,904	3,077
**19**	2240	92.64	168	93.78	24	95.06	6	98.65	5,899,878	2,438
**Total**	33747	92.67	2963	94.04	393	94.96	105	98.66	92,130,833	37,208

Note: For each chromosome, we divided segmental duplication into four subgroups based on SD length and calculated the number of SD and average identity between two SD regions.

Supplementary Table S20. Identification of segmental duplications in the PN40024 genome.

chr	1-5k	Mean identity between SD regions	5-10k	Mean identity between SD regions	10-20k	Mean identity between SD regions	>20k	Mean identity between SD regions	Duplicated region length (bp)[a]	Total number of SD
**1**	1,850	92.80	105	93.09	8	93.55	0	N/A	4,126,638	1,963
**2**	1,724	92.69	100	93.67	7	94.01	1	97.29	3,904,094	1,832
**3**	2,016	92.85	106	93.41	10	93.20	0	N/A	4,509,138	2,132
**4**	1,974	92.91	110	93.47	7	94.12	0	N/A	4,347,417	2,091
**5**	2,366	93.00	129	93.16	6	94.26	0	N/A	5,164,799	2,501
**6**	1,534	92.90	89	93.28	7	94.47	0	N/A	3,419,765	1,630
**7**	1,674	92.93	65	93.24	5	93.41	0	N/A	3,510,930	1,744
**8**	1,551	92.85	92	93.66	4	92.18	0	N/A	3,422,052	1,647
**9**	1,969	93.06	119	93.71	12	94.03	2	97.09	4,522,155	2,102
**10**	1,800	92.73	86	93.56	11	92.85	0	N/A	3,958,669	1,897
**11**	1,783	92.66	99	93.93	7	93.77	0	N/A	3,951,589	1,889
**12**	2,192	92.83	111	93.46	6	93.61	0	N/A	4,729,908	2,309
**13**	2,151	92.81	114	93.64	7	94.03	0	N/A	4,637,554	2,272
**14**	2,685	92.86	145	93.47	15	92.99	0	N/A	5,991,979	2,845
**15**	2,105	92.90	118	93.04	10	93.55	0	N/A	4,703,077	2,233
**16**	2,150	92.87	117	93.22	13	94.35	0	N/A	4,809,973	2,280
**17**	1,233	92.82	100	93.49	6	93.40	0	N/A	2,968,114	1,339
**18**	2,463	92.76	119	93.12	8	93.07	0	N/A	5,286,253	2,590
**19**	2,438	93.00	113	93.28	5	93.14	0	N/A	5,133,121	2,556
**Total**	37,658	92.85	2,037	93.42	154	93.58	3	97.19	83,097,225	39,852

a: Duplicated region length is calculated by taking the sum of the length of all identified pairwise alignments.

Supplementary Table S21. Identification of segmental duplications in the Cabernet Sauvignon genome.

chr	1-5k	Mean identity between SD regions	5-10k	Mean identity between SD regions	10-20k	Mean identity between SD regions	>20k	Mean identity between SD regions	Duplicated region length (bp)	Total number of SD
**1**	1,438	92.69	122	94.04	15	93.26	0	N/A	3,707,377	1,575
**2**	2,819	92.63	217	93.51	23	93.91	2	98.41	7,085,174	3,061
**3**	3,038	92.68	236	93.72	28	94.20	2	98.675	7,714,360	3,304
**4**	1,581	92.78	123	93.48	15	94.87	0	N/A	3,960,241	1,719
**5**	2,447	92.77	227	94.37	29	93.59	1	97.96	6,568,496	2,704
**6**	1,857	92.77	123	94.01	20	94.54	0	N/A	4,600,987	2,000
**7**	2,213	92.77	137	93.77	24	93.34	0	N/A	5,378,134	2,374
**8**	2,072	92.52	170	93.49	23	93.39	3	99.05	5,375,212	2,268
**9**	3,294	92.77	237	93.83	38	93.57	1	98.93	8,279,844	3,570
**10**	2,292	92.53	167	94.01	30	94.21	0	N/A	5,793,276	2,489
**11**	2,579	92.60	188	93.76	20	93.05	0	N/A	6,286,153	2,787
**12**	2,570	92.78	214	93.89	25	93.69	0	N/A	6,550,917	2,809
**13**	2,525	92.88	203	94.11	35	93.08	0	N/A	6,481,859	2,763
**14**	3,317	92.54	262	93.52	53	93.89	0	N/A	8,632,803	3,632
**15**	3,126	92.78	241	93.60	39	93.64	0	N/A	8,002,779	3,406
**16**	3,288	92.70	245	93.73	24	96.22	2	99.08	8,212,355	3,559
**17**	1,601	92.53	147	93.88	21	93.70	1	98.2	4,240,042	1,770
**18**	3,681	92.62	289	93.79	29	93.24	1	99.51	9,088,921	4,000
**19**	3,359	92.72	238	93.83	42	93.67	1	99.49	8,286,106	3,640
**Total**	49,097	92.69	3,786	93.81	533	93.84	14	98.81	124,245,036	53,430

Supplementary Table S22. Identification of segmental duplications in the Chardonnay genome.

chr	1-5k	Mean identity between SD regions	5-10k	Mean identity between SD regions	10-20k	Mean identity between SD regions	>20k	Mean identity between SD regions	Duplicated region length (bp)	Total number of SD
**1**	1,708	92.81	146	94.04	19	95.39	5	97.91	4,632,877	1,878
**2**	1,573	92.59	155	93.96	13	95.02	0	N/A	4,216,978	1,741
**3**	2,071	92.72	143	94.14	14	95.95	2	98.95	5,188,574	2,230
**4**	1,964	92.80	138	93.96	17	94.99	2	99.19	4,918,079	2,121
**5**	2,641	92.76	246	94.45	33	95.86	7	98.02	7,321,399	2,927
**6**	1,461	92.71	109	93.69	15	95.68	3	98.21	3,762,775	1,588
**7**	1,411	92.77	107	94.57	16	95.17	3	98.96	3,649,032	1,537
**8**	1,563	92.88	138	94.31	18	95.69	4	97.69	4,250,908	1,723
**9**	1,889	92.81	139	93.97	22	95.53	4	99.18	4,999,950	2,054
**10**	1,791	92.58	123	94.00	15	95.59	2	98.71	4,482,415	1,931
**11**	1,623	92.69	145	94.03	21	95.30	3	98.87	4,412,991	1,792
**12**	2,104	92.86	152	94.03	26	95.85	3	98.52	5,417,146	2,285
**13**	2,246	92.83	202	94.29	21	96.33	8	98.36	6,141,837	2,477
**14**	2,968	92.78	270	94.24	40	95.60	6	98.65	8,121,344	3,284
**15**	2,112	92.70	180	93.82	20	94.60	1	99.24	5,516,552	2,313
**16**	2,348	92.71	165	93.74	35	96.46	5	98.51	6,155,385	2,553
**17**	1,372	92.60	114	93.86	19	94.90	3	99.11	3,654,573	1,508
**18**	2,166	92.66	165	94.48	18	95.35	3	97.96	5,635,377	2,352
**19**	2,398	92.82	218	94.33	36	95.25	9	98.35	6,796,588	2,661
**Total**	37,409	92.74	3,055	94.10	418	95.50	73	98.58	99,274,780	40,955

Supplementary Table S23. Identification of segmental duplications in the *V. riparia* genome.

chr	1-5k	Mean identity between SD regions	5-10k	Mean identity between SD regions	10-20k	Mean identity between SD regions	>20k	Mean identity between SD regions	Duplicated region length (bp)	Total number of SD
**1**	1,744	92.60	140	94.43	11	94.65	1	98.93	4,355,487	1,896
**2**	1,644	92.65	117	93.97	11	94.55	1	99.16	3,939,203	1,773
**3**	1,847	92.56	124	93.53	12	93.95	0	N/A	4,480,780	1,983
**4**	1,700	92.63	154	94.15	15	94.22	2	97.79	4,395,624	1,871
**5**	1,721	92.56	146	94.35	14	96.26	0	N/A	4,519,692	1,881
**6**	1,744	92.54	157	93.75	10	95.57	1	98.93	4,409,787	1,912
**7**	2,076	92.58	160	94.16	16	95.07	0	N/A	5,182,568	2,252
**8**	1,314	92.54	119	94.24	6	95.72	0	N/A	3,326,712	1,439
**9**	1,795	92.73	133	94.10	15	95.01	1	98.41	4,586,184	1,944
**10**	1,768	92.62	121	94.44	10	96.03	1	98.17	4,395,784	1,900
**11**	1,478	92.65	128	94.35	10	94.87	0	N/A	3,761,525	1,616
**12**	1,834	92.59	149	94.04	17	94.97	1	97.43	4,588,056	2,001
**13**	2,206	92.69	195	93.88	15	94.24	3	97.34	5,754,681	2,419
**14**	2,283	92.66	181	93.95	15	94.37	2	97.44	5,698,745	2,481
**15**	2,043	92.64	180	93.70	28	96.29	2	98.31	5,474,773	2,253
**16**	2,149	92.53	189	94.09	12	94.40	2	99.36	5,605,266	2,352
**17**	1,604	92.35	129	94.21	18	96.31	1	98.17	4,118,295	1,752
**18**	3,190	92.56	244	94.04	15	92.90	1	97.58	7,883,432	3,450
**19**	2,244	92.60	173	93.99	26	94.69	2	96.87	5,726,571	2,445
**Total**	36,384	92.59	2,939	94.07	276	94.95	21	98.13	92,203,165	39,620

Supplementary Table S24. The collinearity among the grapevine genomes for genes embedded within SDs.

	**PN40024**	**Cabernet Sauvignon**	**Chardonnay**	***V. labrusca***	***V. riparia***
Total	857	1981	3349	3049	2603
All Shared	214	471	934	942	1238
Sub Shared[a]	270	441	428	115	130
Genome-specific	373	1069	1987	1992	1235

[a]Sub Shared: genes that were shared by either the cultivated (PN40024, Cab, and Char) or wild (Vlab and Vrip) genomes.

Supplementary Table S25. Ka/Ks ratio calculations for segmental duplication derived genes.

Genome	Gene count (Ka/Ks<1)	Gene count (Ka/Ks>1)
**PN40024**	115	31
**Cabernet Sauvignon**	493	94
**Chardonnay**	1022	198
V. labrusca	650	166
V. riparia	758	113

Supplementary Table S26. Gene cluster analysis among the five grapevine genomes from OrthoFinder. For each category, the numbers outside the brackets are the number of gene cluster; inside the brackets are the number of gene.

species	Protein number	Gene cluster number	Singletons	Gene clusters (genes) shared by all	Unique gene clusters (genes)	Proportion of genome specific genes (%)	Unique gene number in cultivated grapes	Unique gene number in wild grapes
**PN40024**	29,927	21,438	2,561	14,894 (19,049)	126 (347)	9.72	435 (591)	NA
**Cabernet Sauvignon**	36,660	22,285	2,881	14,894 (21,888)	376 (943)	10.43	435 (765)	NA
**Chardonnay**	34,458	21,960	1,576	14,894 (19,753)	230 (1,194)	8.04	435 (634)	NA
V. riparia	37,133	21,666	2,081	14,894 (20,573)	626 (2,826)	13.21	NA	416 (1,016)
V. labrusca	35,915	22,051	1,656	14,894 (19,915)	410 (1,645)	9.19	NA	416 (705)

Supplementary Table S27. GO enrichment analysis of genome-specific gene families from the five grapevines.

Species	GOTerm	Description	Gene Ratio	P value	p.adjust
**PN40024**					
	GO:0016747	transferase activity, transferring acyl groups other than amino-acyl groups	51/712	5.69E-30	2.55E-27
	GO:0009058	biosynthetic process	45/712	4.90E-27	1.10E-24
	GO:0016746	transferase activity, transferring acyl groups	39/712	1.50E-23	2.24E-21
	GO:0007064	mitotic sister chromatid cohesion	6/712	7.08E-05	0.00793233
**Cabernet Sauvignon**					
	GO:0015074	DNA integration	45/1431	8.68E-06	0.00626382
	GO:0006401	RNA catabolic process	7/1431	0.00013759	0.03338882
	GO:0010333	terpene synthase activity	22/1431	0.00018188	0.03338882
	GO:0019843	rRNA binding	10/1431	0.0002266	0.03338882
	GO:0016874	ligase activity	18/1431	0.00023122	0.03338882
	GO:0004812	aminoacyl-tRNA ligase activity	15/1431	0.00031428	0.03769127
	GO:0006418	tRNA aminoacylation for protein translation	14/1431	0.00036543	0.03769127
	GO:0000027	ribosomal large subunit assembly	8/1431	0.00046474	0.04194278
**Chardonnay**					
	GO:0004523	RNA-DNA hybrid ribonuclease activity	161/1203	7.19E-109	3.01E-106
	GO:0090502	RNA phosphodiester bond hydrolysis, endonucleolytic	161/1203	1.67E-101	3.49E-99
V. labrusca					
	GO:0046983	protein dimerization activity	102/1900	1.27E-20	5.92E-18
	GO:0008234	cysteine-type peptidase activity	58/1900	3.21E-16	7.51E-14
	GO:0004190	aspartic-type endopeptidase activity	42/1900	2.86E-13	4.46E-11
	GO:0010333	terpene synthase activity	29/1900	7.79E-07	9.12E-05

Supplementary Table S27. Continued.

Species	GOTerm	Description	Gene Ratio	P value	p.adjust
V. riparia					
	GO:0015074	DNA integration	138/1687	1.16E-64	6.57E-62
	GO:0004523	RNA-DNA hybrid ribonuclease activity	11/1687	1.41E-06	0.00039982
	GO:0043565	sequence-specific DNA binding	47/1687	4.69E-05	0.00886132
	GO:0009767	photosynthetic electron transport chain	7/1687	8.71E-05	0.01234081
	GO:0140326	ATPase-coupled intramembrane lipid transporter activity	7/1687	0.00013382	0.01517554
	GO:0019684	photosynthesis, light reaction	8/1687	0.00029168	0.02442208
	GO:0009269	response to desiccation	5/1687	0.00031914	0.02442208
	GO:0070652	HAUS complex	6/1687	0.00034458	0.02442208
	GO:0003712	transcription coregulator activity	13/1687	0.00051238	0.03228009

Supplementary Table S28. Gene family evolution calculated by CAFE5.

Species	Expansions	Contractions	Rapidly evolving	Rapidly expanded	Rapidly contracted
**PN40024**	806	1385	150	36	114
**Cabernet Sauvignon**	2319	1144	227	200	27
**Chardonnay**	934	1954	66	47	19
**Cultivated**	70	165	3	2	1
V. riparia	1576	1169	191	147	44
V. labrusca	774	1638	103	62	41
**Wild**	328	903	31	24	7

Supplementary Table S29. Functional analysis on rapidly amplified gene families in wild grapes

ID	Description	Gene Ratio	P value	p.adjust	Count
**GO:0005544**	calcium-dependent phospholipid binding	45/144	4.20E-95	2.31E-93	45
**GO:0005509**	calcium ion binding	45/144	3.11E-51	8.55E-50	45
**GO:0050832**	defense response to fungus	18/144	8.67E-27	1.59E-25	18
**GO:0030130**	clathrin coat of trans-Golgi network vesicle	12/144	8.25E-24	9.08E-23	12
**GO:0030132**	clathrin coat of coated pit	12/144	8.25E-24	9.08E-23	12
**GO:0009409**	response to cold	14/144	5.47E-22	5.01E-21	14
**GO:0009414**	response to water deprivation	14/144	1.19E-20	9.34E-20	14
**GO:0009408**	response to heat	14/144	3.34E-19	2.30E-18	14
**GO:0005198**	structural molecule activity	12/144	2.45E-17	1.50E-16	12
**GO:0009651**	response to salt stress	14/144	1.09E-16	6.00E-16	14
**GO:0005576**	extracellular region	18/144	1.53E-10	7.67E-10	18
**GO:0016192**	vesicle-mediated transport	12/144	7.45E-10	3.42E-09	12
**GO:0006898**	receptor-mediated endocytosis	5/144	2.08E-08	8.79E-08	5
**GO:0006952**	defense response	8/144	3.08E-08	1.14E-07	8
**GO:0006886**	intracellular protein transport	12/144	3.10E-08	1.14E-07	12
**GO:0005543**	phospholipid binding	5/144	3.50E-07	1.20E-06	5
**GO:0005545**	1-phosphatidylinositol binding	4/144	2.79E-06	9.02E-06	4
**GO:0048268**	clathrin coat assembly	4/144	3.57E-06	1.09E-05	4
**GO:0030136**	clathrin-coated vesicle	4/144	5.60E-06	1.62E-05	4
**GO:0030276**	clathrin binding	4/144	2.63E-05	7.23E-05	4
**GO:0030599**	pectinesterase activity	5/144	4.84E-05	1.16E-04	5
**GO:0042545**	cell wall modification	5/144	4.84E-05	1.16E-04	5
**GO:0045330**	aspartyl esterase activity	5/144	4.84E-05	0.00011566	5
**GO:0045490**	pectin catabolic process	5/144	8.39E-05	1.92E-04	5
**GO:0004857**	enzyme inhibitor activity	5/144	1.29E-04	0.00028384	5
**GO:0043086**	negative regulation of catalytic activity	5/144	1.45E-04	0.00030633	5
**GO:0030659**	cytoplasmic vesicle membrane	2/144	1.90E-03	0.0038683	2
**GO:0009506**	plasmodesma	5/144	0.00261041	0.00512759	5

Supplementary Table S29. Continued.

ID	Description	Gene Ratio	P value	p.adjust	Count
**GO:0005905**	clathrin-coated pit	2/144	0.00356872	0.00676826	2
**GO:0010427**	abscisic acid binding	2/144	0.00763845	0.01400382	2
**GO:0004864**	protein phosphatase inhibitor activity	2/144	0.00833322	0.01478474	2
**GO:0032515**	negative regulation of phosphoprotein phosphatase activity	2/144	0.00905526	0.01556373	2
**GO:0009738**	abscisic acid-activated signaling pathway	2/144	0.00980424	0.0163404	2
**GO:0015171**	amino acid transmembrane transporter activity	2/144	0.01484452	0.0240132	2
**GO:0003333**	amino acid transmembrane transport	2/144	0.01770099	0.02781584	2
**GO:0038023**	signaling receptor activity	2/144	0.02293005	0.03503202	2
**GO:0031410**	cytoplasmic vesicle	2/144	0.02517835	0.03742728	2

Supplementary Table S30. Functional analysis on rapidly contracted gene families in wild grapes

ID	Description	Gene Ratio	P value	p.adjust	Count
**GO:0031982**	vesicle	9/14	6.07E-29	9.72E-28	9
**GO:0006898**	receptor-mediated endocytosis	9/14	1.38E-27	1.10E-26	9
**GO:0072583**	clathrin-dependent endocytosis	9/14	2.25E-25	1.20E-24	9
**GO:0030276**	clathrin binding	9/14	2.76E-24	1.10E-23	9
**GO:0043231**	intracellular membrane-bounded organelle	9/14	3.19E-15	1.02E-14	9
**GO:0000159**	protein phosphatase type 2A complex	2/14	3.78E-05	0.00010071	2
**GO:0019888**	protein phosphatase regulator activity	2/14	5.97E-05	0.0001365	2
**GO:0043666**	regulation of phosphoprotein phosphatase activity	2/14	9.41E-05	0.00018827	2
**GO:0004722**	protein serine/threonine phosphatase activity	2/14	0.00112378	0.00199783	2
**GO:0006470**	protein dephosphorylation	2/14	0.00340745	0.00545192	2
**GO:0004527**	exonuclease activity	1/14	0.01334142	0.01940571	1
**GO:0000049**	tRNA binding	1/14	0.02765539	0.03543647	1
**GO:0008033**	tRNA processing	1/14	0.02879214	0.03543647	1

Supplementary Table S31. Functional analysis on rapidly amplified gene families in PN40024.

ID	Description	Gene Ratio	P value	p.adjust
**GO:0051259**	protein complex oligomerization	34/335	5.44E-61	4.25E-59
**GO:0016844**	strictosidine synthase activity	30/335	3.55E-55	1.39E-53
**GO:0000302**	response to reactive oxygen species	34/335	7.83E-54	2.04E-52
**GO:0043621**	protein self-association	34/335	6.20E-50	1.21E-48
**GO:0008422**	beta-glucosidase activity	30/335	1.08E-44	1.68E-43
**GO:0009651**	response to salt stress	37/335	1.95E-39	2.54E-38
**GO:0009408**	response to heat	34/335	7.71E-39	8.59E-38
**GO:0008757**	S-adenosylmethionine-dependent methyltransferase activity	33/335	4.90E-36	4.78E-35
**GO:0045551**	cinnamyl-alcohol dehydrogenase activity	19/335	4.52E-35	3.92E-34
**GO:0004032**	alditol:NADP+ 1-oxidoreductase activity	19/335	8.91E-34	6.32E-33
**GO:0008106**	alcohol dehydrogenase (NADP+) activity	19/335	8.91E-34	6.32E-33
**GO:0032259**	methylation	41/335	1.09E-32	7.11E-32
**GO:0009809**	lignin biosynthetic process	19/335	3.78E-31	2.27E-30
**GO:0051082**	unfolded protein binding	34/335	6.54E-30	3.65E-29
**GO:0008168**	methyltransferase activity	43/335	7.10E-30	3.69E-29
**GO:0006457**	protein folding	34/335	1.43E-28	6.98E-28
**GO:0009696**	salicylic acid metabolic process	13/335	3.57E-24	1.39E-23
**GO:0080030**	methyl indole-3-acetate esterase activity	13/335	3.57E-24	1.39E-23
**GO:0080031**	methyl salicylate esterase activity	13/335	3.57E-24	1.39E-23

Supplementary Table S31. Continued

ID	Description	GeneRatio	BgRatio	pvalue	p.adjust
**GO:0080032**	methyl jasmonate esterase activity	13/335	13/20939	3.57E-24	1.39E-23
**GO:0009694**	jasmonic acid metabolic process	13/335	14/20939	4.92E-23	1.83E-22
**GO:0004553**	hydrolase activity, hydrolyzing O-glycosyl compounds	38/335	306/20939	7.75E-23	2.75E-22
**GO:0009058**	biosynthetic process	30/335	175/20939	2.37E-22	8.04E-22
**GO:0016616**	oxidoreductase activity, acting on the CH-OH group of donors, NAD or NADP as acceptor	24/335	135/20939	1.72E-18	5.57E-18
**GO:0000463**	maturation of LSU-rRNA from tricistronic rRNA transcript (SSU-rRNA, 5.8S rRNA, LSU-rRNA)	12/335	29/20939	9.40E-15	2.93E-14
**GO:0005783**	endoplasmic reticulum	30/335	368/20939	3.00E-13	9.00E-13
**GO:0042773**	ATP synthesis coupled electron transport	7/335	12/20939	1.86E-10	5.38E-10
**GO:0010951**	negative regulation of endopeptidase activity	9/335	31/20939	9.12E-10	2.54E-09
**GO:0008137**	NADH dehydrogenase (ubiquinone) activity	7/335	21/20939	2.42E-08	6.50E-08
**GO:0022625**	cytosolic large ribosomal subunit	12/335	121/20939	5.68E-07	1.48E-06
**GO:0003954**	NADH dehydrogenase activity	5/335	17/20939	5.38E-06	1.35E-05
**GO:0009060**	aerobic respiration	5/335	18/20939	7.35E-06	1.79E-05
**GO:0008033**	tRNA processing	7/335	54/20939	2.34E-05	5.53E-05
**GO:0000049**	tRNA binding	6/335	49/20939	0.0001256	0.00028814
**GO:0043531**	ADP binding	16/335	364/20939	0.00027777	0.00061902
**GO:0005773**	vacuole	8/335	143/20939	0.00216746	0.00469617
**GO:0051537**	2 iron, 2 sulfur cluster binding	4/335	35/20939	0.00227832	0.00480295
**GO:0000045**	autophagosome assembly	3/335	26/20939	0.00803383	0.0164905
**GO:0003735**	structural constituent of ribosome	12/335	349/20939	0.01089838	0.02125502
**GO:0005747**	mitochondrial respiratory chain complex I	3/335	29/20939	0.01090001	0.02125502
**GO:0015979**	photosynthesis	5/335	86/20939	0.01233898	0.02347416
**GO:0009579**	thylakoid	4/335	69/20939	0.02467225	0.04581989

Supplementary Table S32. Functional analysis on rapidly contracted gene families in PN40024.

ID	Description	Gene Ratio	P value	p.adjust
**GO:0005544**	calcium-dependent phospholipid binding	31/340	2.01E-48	3.40E-46
**GO:0009409**	response to cold	27/340	4.33E-34	3.66E-32
**GO:0009664**	plant-type cell wall organization	21/340	3.84E-33	2.16E-31
**GO:0009414**	response to water deprivation	29/340	6.19E-33	2.62E-31
**GO:0008146**	sulfotransferase activity	17/340	2.77E-28	9.37E-27
**GO:0009408**	response to heat	26/340	3.41E-26	9.61E-25
**GO:0009651**	response to salt stress	27/340	1.10E-24	2.65E-23
**GO:0005985**	sucrose metabolic process	13/340	4.34E-24	8.15E-23
**GO:0140326**	ATPase-coupled intramembrane lipid transporter activity	13/340	5.99E-23	1.01E-21
**GO:0045332**	phospholipid translocation	12/340	2.77E-22	4.25E-21
**GO:0005509**	calcium ion binding	32/340	7.22E-19	9.38E-18
**GO:0010608**	posttranscriptional regulation of gene expression	10/340	1.12E-18	1.35E-17
**GO:0003843**	1,3-beta-D-glucan synthase activity	9/340	6.96E-16	6.92E-15
**GO:0005576**	extracellular region	35/340	1.59E-14	1.41E-13
**GO:0003746**	translation elongation factor activity	12/340	7.24E-14	5.82E-13
**GO:0071555**	cell wall organization	21/340	4.29E-12	3.15E-11
**GO:0005375**	copper ion transmembrane transporter activity	7/340	3.22E-11	2.27E-10
**GO:0008610**	lipid biosynthetic process	9/340	3.72E-10	2.51E-09
**GO:0035434**	copper ion transmembrane transport	7/340	1.61E-09	1.05E-08
**GO:0003924**	GTPase activity	15/340	3.08E-07	1.93E-06
**GO:0008033**	tRNA processing	8/340	2.42E-06	1.46E-05
**GO:0006400**	tRNA modification	6/340	3.89E-06	2.27E-05
**GO:0003729**	mRNA binding	12/340	1.24E-05	7.01E-05
**GO:0019752**	carboxylic acid metabolic process	5/340	1.80E-05	9.82E-05

Supplementary Table S33. Summary of NLR gene families in the five grapevine genomes.

	**PN40024**	**Cabernet Sauvignon**	**Chardonnay**	***V. riparia***	***V. labrusca***
**NB-ARC**	348	735	662	884	911
**TIR**	77	159	189	268	313
**CC**	3867	4624	4558	4686	5211
**CCr**	17	7	5	9	11
**LRR**	566	911	774	983	956
**TNLs (TIR+NB)**	28	92	124	206	250
**TNLs (TIR only)**	49	67	65	62	63
**CNLs (CC+NB)**	207	443	358	478	474
**RNLs (CCr+NB)**	14	7	5	8	9
**RNLs (CCr only)**	3	0	0	1	2
**NLs (NB+LRR)**	29	52	40	37	62
**NLs (NB only)**	70	141	135	155	116
**Total**	400	802	727	947	976

Supplementary Table S34. Summary of LTR annotation in the five grapevine genomes

Species	Total number	Total length (bp)	Percentage of genome (%)	Ty1/Copia (count/length)	Ty3/Gypsy (count/length)
**PN40024**	134211	113610559	23.36	57118/49203916	52519/50788974
**Cabernet Sauvignon**	217452	193502935	32.71	82351/67162090	78242/84065711
**Chardonnay**	178470	133660429	27.27	82114/58399298	57376/50928759
V. labrusca	217909	135246080	26.93	40629/18133666	65875/61608669
V. riparia	156330	130141562	26.02	69453/52896082	55709/59719157

Supplementary Table S35. Summary of MITE annotation in the five grapevine genomes.

**Species** [a]	**Cabernet Sauvignon**	**Chardonnay**	**PN40024**	***V. labrusca***	***V. riparia***
**DTC count**	102277	77916	91894	85465	87937
**DTC length**	29400428	27314636	29115468	26597729	26245048
**DTM count**	166935	142684	160185	148645	168290
**DTM length**	51503586	47279540	57676526	50204996	53435347
**DTH count**	54658	41038	38400	39559	38764
**DTH length**	18749897	13818778	13700240	13032908	14870469
**DTT count**	24051	13251	13934	11271	22368
**DTT length**	6135521	4464853	5387899	2674850	6436541
**Total count**	347921	274889	304413	284940	317359
**Total length**	105789432	92877807	105880133	92510483	100987405

a: DTC, CACTA; DTM, Mutator; DTH, PIF/Harbinger and DTT, Tc1/mariner.

Supplementary Table S36. Summary of TE insertions (LTR and MITE) around collinear gene regions.

	PN40024	Cabernet Sauvignon	Chardonnay	*V. riparia*	*V. labrusca*
**Collinear Gene Count**	12155	12155	12155	12155	12155
**LTR insertion within 1kb upstream of collinear genes**	1651	2482	2794	3506	3161
**LTR insertion within genic regions of collinear genes**	2981	3386	3617	5883	3732
**LTR insertion within 1kb downstream of collinear genes**	1537	2169	2099	3306	2583
**MITE insertion within 1kb upstream of collinear genes**	6305	6802	5911	6279	5786
**MITE insertion within genic regions of collinear genes**	6549	7144	6901	8556	6763
**MITE insertion within 1kb downstream of collinear genes**	5651	6050	5208	5632	5027
